# Survival and multi-omics analysis of immunotherapy-based conversion treatment for initially unresectable gastric cancer

**DOI:** 10.3389/fimmu.2025.1753749

**Published:** 2026-01-13

**Authors:** Song Li, Qian Xu, Wenbin Yu, Zimin Liu, Xiaohan Cui, Meng Wei, Duanbo Shi, Wen Zhao, Xinyu Song, Di Zhang, Zhaodi Nan, Jiahui Chu, Shuang Wang, Shulun Nie, Xin Dai, Lian Liu

**Affiliations:** 1 Department of Medical Oncology, Qilu Hospital, Cheeloo College of Medicine, Shandong University, Jinan, Shandong, China; 2 Department of General Surgery, Qilu Hospital, Cheeloo College of Medicine, Shandong University, Jinan, Shandong, China; 3 Department of Medical Oncology, The Affiliated Hospital of Qingdao University, Qingdao, Shandong, China; 4 Department of Pathology, Qilu Hospital, Cheeloo College of Medicine, Shandong University, Jinan, Shandong, China; 5 Department of Pharmacy, Qilu Hospital, Cheeloo College of Medicine, Shandong University, Jinan, Shandong, China; 6 Department of Medical Oncology, Shandong Provincial Hospital of Traditional Chinese Medicine, Jinan, Shandong, China

**Keywords:** conversion treatment, gastric cancer, immunotherapy, initially unresectable, multi-omics

## Abstract

**Background:**

Immune checkpoint inhibitors (ICIs) have demonstrated efficacy in the treatment of resectable and advanced gastric cancer. However, their effectiveness in initially unresectable gastric cancer remains unclear. This study aims to evaluate the efficacy and elucidate the molecular mechanisms of conversion therapy utilizing ICIs in patients with initially unresectable gastric cancer.

**Methods:**

This retrospective cohort study included patients with initially unresectable locally advanced or oligometastatic gastric cancer treated with ICIs-based therapy for conversion purposes. The primary endpoints were rates of complete pathological response (pCR), major pathological response (MPR), and R0 resection. Secondary endpoints included overall survival (OS), disease-free survival (DFS), event-free survival (EFS) and safety. A multi-omics analysis was performed on the tumor samples.

**Results:**

A total of 73 patients were analyzed, with 75.3% at stage cIV. The median EFS and OS were 26.1 and 41.3 months, respectively. 82.2% of the patients underwent radical surgery and achieved a median EFS of 30.9 months. The pCR and MPR rates were 24.7% and 37.0%, respectively. R0 resection was associated with superior survival, and MPR further enhanced efficacy. The incidence of adverse effects and postoperative complications were within acceptable limits. KMT2D mutations were associated with improved outcomes and enhanced immune responses, whereas tumor cytochrome P450 was linked to resistance.

**Conclusion:**

ICIs-based conversion therapy demonstrates promising survival benefits and manageable safety profiles for patients with unresectable locally advanced or oligometastatic gastric cancer, and provides real-world evidence for a potential treatment strategy. Additionally, multi-omics findings have revealed drug resistance mechanisms and potential biomarkers of efficacy.

## 1 Introduction

Gastric cancer (GC) is the fifth most common and fourth deadliest cancer globally, causing about one million new cases and 700,000 deaths annually (1). Radical resection is the main treatment to cure and extend survival, but many patients present with unresectable disease due to local invasion, non-regional lymph node metastasis, liver metastasis, and peritoneal dissemination (2). For these patients, palliative therapy is standard but typically results in poor survival outcomes of 12 to 16 months (3). Neoadjuvant therapy has been established as the standard treatment for resectable locally advanced GC (4). The potential of conversion therapy is still under investigation, facing challenges such as low conversion and R0 resection rates (5). In the AIO-FLOT3 study, patients with limited metastatic disease showed better survival following conversion chemotherapy and surgery, but only 60% underwent surgery, with just 43.3% achieving R0 resection (6).

Immune checkpoint inhibitors (ICIs) enhance T-cell responses and prevent cancer cells from evading immune detection (7–9), offering promising survival benefits as first-line treatments for unresectable locally advanced and metastatic GC. However, overall survival (OS) in these randomized controlled trials (RCTs) remains below 15 months (10). Phase III RCTs in the perioperative setting, such as KEYNOTE-585 (11), MATTERHORN (12), and DRAGON-IV (13), suggest that adding ICIs to neoadjuvant chemotherapy improves pathological complete response (pCR) rates and disease-free survival (DFS). However, these trials included only 4%, 3%, and 1% of cT4b patients, respectively, and no cM1 patients (11–13), limiting their applicability to cIVA and metastatic cIVB GC patients. To date, the treatment strategies for these patients often reference those established for advanced metastatic disease (14, 15), and research on ICIs in conversion therapy for initially unresectable GC is sparse. Additionally, while perioperative immunotherapy studies often assess traditional biomarkers like programmed death-ligand 1 (PD-L1) expression (16), microsatellite instability (MSI) status (17), tumor mutational burden (TMB) (18), a comprehensive high-throughput analysis of efficacy prediction and resistance mechanisms remains underexplored.

In summary, current research on conversion immunotherapy for initially unresectable GC is sparse, necessitating urgent validation in larger cohorts and advanced-stage populations, along with exploration of tumor immune microenvironments through high-throughput genomic and transcriptomic approaches. Consequently, we conducted a real-world retrospective cohort study on ICI-based conversion therapies for unresectable GC, incorporating multi-omics analyses to assess survival outcomes and safety and to identify potential biomarkers for treatment efficacy and resistance mechanisms.

## 2 Materials and methods

### 2.1 Patient inclusion

This single-institution retrospective study, approved by the Medical Ethical Committee (approval number: 2018214) and the Ethics Committee on Scientific Research (approval number: 202107-059) of Shandong University Qilu Hospital, included patients with gastric or gastroesophageal junction adenocarcinoma who were intended to receive conversion therapy from June 1, 2019, to December 31, 2022. Clinical data were collected via the hospital’s electronic medical record system, with follow-up at least every 3 months. All clinical data were de-identified prior to use, and informed consent was waived as approved by the Ethics Committee. The data cutoff for the analyses was November 16, 2023. The inclusion criteria were as follows: 1. pathological confirmation of gastric or gastroesophageal junction adenocarcinoma; 2. initially unresectable locally advanced GC or oligometastatic patients who were considered candidates for conversion therapy by a multidisciplinary team (MDT); and 3. completion of at least two cycles of ICI-containing therapy (ICI + chemotherapy ± antiangiogenetic agents), followed by surgical resection or not. The exclusion criteria were as follows: 1. prior neoadjuvant radiotherapy. 2. Patients with concurrent or recent other malignancies within 5 years. Treatment regimens and the number of cycles were determined primarily by the treating physician in consultation with the MDT, informed by evidence from relevant clinical studies and a comprehensive assessment of the patient’ s underlying conditions and potential drug toxicities.

### 2.2 Study endpoints and assessments

The primary endpoints were rates of pCR, major pathological response (MPR), and R0 resection. Secondary endpoints included OS, DFS, event-free survival (EFS), incidence of any grade or grade ≥3 treatment-related adverse events (TRAEs), and postoperative complications. The exploratory endpoint was the correlation between tumor genetic characteristics and efficacy. Two independent pathologists assessed the pathological response by determining the percentage of residual viable tumor cells in the tumor bed. pCR was defined as the absence of viable tumor cells, whereas MPR was defined as the presence of no more than 10% viable tumor cells. OS was measured from the first treatment dose to death from any cause. DFS was measured from surgery to disease recurrence or death in patients with R0 resection. EFS was measured from the first treatment dose to disease progression, recurrence, or death from any cause. For individuals lost to follow-up, the date of their last visit or follow-up was used for censoring. Adverse events were assessed according to the Common Terminology Criteria for Adverse Events (CTCAE) v5.0, and postoperative complications were graded using the Clavien-Dindo system (19). PD-L1 IHC was performed using the PD-L1 IHC 22C3 pharmDx kit (Dako) on the Dako ASL48 platform, in accordance with manufacturer recommendations.

### 2.3 Genome sequencing and analysis

Our cohort (Qilu cohort, 73 patients) included 58 patients who underwent genomic profiling via whole-exome sequencing at 300× depth (n = 19) or targeted sequencing of 324 core driver genes (n = 39). Analyses of single nucleotide variants (SNVs), insertions and deletions (indels), and copy number variations (CNVs) were performed on genes common to both methods. TMB was quantified as nonsynonymous somatic mutations per megabase, with TMB-H defined as TMB ≥10muts/Mb. MSI was assessed using MSIsensor software (version 0.6). For external validation, targeted sequencing data from an independent cohort (the Qingdao Cohort) of 16 patients with locally advanced GC who received neoadjuvant immunotherapy combined with chemotherapy at the Affiliated Hospital of Qingdao University were used.

### 2.4 Transcriptome sequencing

Transcriptome sequencing of 27 tumor samples was conducted. Differential expression analysis using the limma package identified DEGs based on absolute log2(fold change) > 1 and P-value < 0.01. Pathway enrichment analyses for Gene Ontology (GO), Kyoto Encyclopedia of Genes and Genomes (KEGG), Reactome, and cancer hallmarks were performed using GSEA. Single-sample pathway enrichment analysis was conducted using the GSVA package. Immune cell infiltration abundance was estimated in each sample using the R packages (20), CIBERSORT (21), and MCP-counter (22). The Immunophenogram tool was used to assess immunophenotypes, focusing on major histocompatibility complexes (MHC), immune checkpoints, effector cells, and suppressor cells (23).

### 2.5 Statistical analysis

Statistical analyses were performed using R (v3.6.1), GraphPad Prism (v9.5), and SPSS (v26). Categorical variables were presented as percentages with 95% confidence intervals (CIs), and intergroup differences were assessed via Chi-square or Fisher’s exact tests. Continuous variables, shown as medians and quartiles, were compared using the unpaired t-test or Mann-Whitney U test. Correlations between continuous variables were evaluated using Spearman’s rank correlation coefficient. OS, DFS, and EFS were estimated using the Kaplan-Meier method and compared using the log-rank test. Univariable and multivariable Cox regression analyses were used to determine the hazard ratios (HR) and their 95% CIs. All P-values were two-sided, with significance set at P < 0.05.

### 2.6 Multivariable modelling and sensitivity analyses

Multivariable Cox proportional hazards models were employed to assess the independent associations of variables with survival outcomes based on classical statistical assumptions. Covariate selection for the final model was guided by a combination of statistical significance and clinical relevance. Specifically, variables that showed an association with OS or DFS in univariable analysis (using a threshold of P < 0.05) were considered candidates. Furthermore, established biomarkers of biological significance in the context of immunotherapy (e.g., MMR status), even if not statistically significant in univariable analysis, were included to enhance the clinical interpretability of the model. To mitigate the concern of multicollinearity, the variance inflation factor (VIF) was calculated for each variable included in the multivariable models. A conventional threshold of 5 was used to indicate significant collinearity among the multiple indicators in the model. To further test the robustness of key findings, sensitivity analyses were conducted. This involved sequentially refitting the multivariable Cox models while excluding specific covariates of interest (namely, MMR status, Lauren classification, and PD-L1 expression) to observe the stability of the effect estimates for TMB.

## 3 Results

### 3.1 Patient characteristics

**Figure 1:**
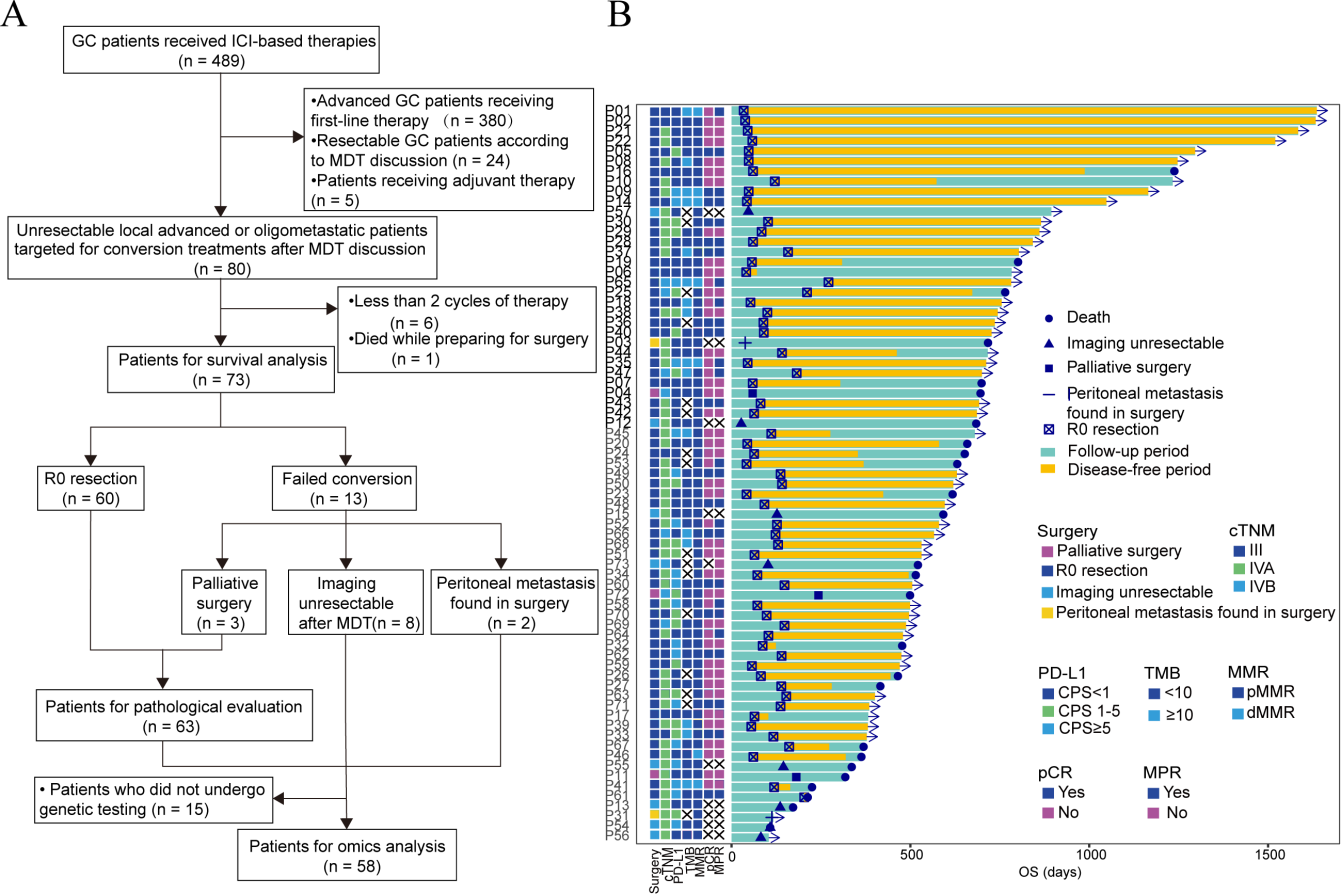
Overview of patient enrollment and follow-up. **(A)** Patients’ treatment process. Patients with unresectable locally advanced or oligometastatic gastric cancer (GC) received immune checkpoint inhibitors (ICIs) + chemotherapy ± antiangiogenic agents for conversion purpose, followed by surgical resection. Tumor biopsy samples were used for multi-omics analysis. **(B)** Swimmer plot depicting individual trajectories of the 73 patients, providing a visual representation of treatment duration and outcomes. CPS, combined positive score; ICI, immune checkpoint inhibitor; MDT, multidisciplinary team; MMR, mismatch repair; PD-L1, programmed death-ligand 1; TMB, tumor mutational burden.

A total of 489 GC patients who received ICI-based therapies were screened. Of these, we excluded 380 patients aimed with first-line palliative therapy, 24 with neoadjuvant therapy, and 5 with adjuvant therapy. According to the predefined analysis plan, patients with less than 2 cycles of treatment (n = 6) and those who died while preparing for surgery (n = 1) were excluded. Finally, 73 patients initially deemed unresectable by the MDT were included in the analysis. All 73 patients were assessed for OS, 63 for pathological response, and 58 for multi-omics analysis (**Figure 1A**). Among the 73 patients, 58 had adenocarcinomas, 14 had signet-ring cell carcinomas, and one had adenosquamous carcinoma. Under clinical staging, 18 (24.7%) were cIII, 47 (64.4%) were cIVA, and eight (10.9%) were cIVB, which included four peritoneal, two hepatic, and two ovarian oligometastases. PD-L1 CPS was ≥1 in 36 (49.3%) patients, ≥5 in 18 (24.7%), and ≥10 in 11 (15.1%). Additionally, eight patients (11.0%) were HER2 positive, seven (9.6%) were dMMR, 16 (21.9%) were TMB-H, and two (2.7%) were EBER-positive (**Table 1**). Patients received a median of 3 (quantile 2–5) cycles of ICI-based conversion therapy, with 24 patients receiving the combined therapy of ICIs and chemotherapy, and 49 receiving the combination of ICIs, chemotherapy, and apatinib (**Table 1**). The detailed distribution of treatment cycles and treatment regimens across the cohort is provided in **Supplementary Table S1**. One HER2-positive patient also received trastuzumab (**Table 1**). The median follow-up was 21.0 months (quantile, 15.8-25.8) (**Figure 1B**).

**Table 1:** Baseline demographic and treatment characteristics.

Variables	All patients (n = 73)	Clinical stage III (n = 18)	Clinical stage IVA (n = 47)	Clinical stage IVB (n = 8)
Gender [n (%)]				
Male	55 (75.3)	16 (88.9)	34 (72.3)	5 (62.5)
Female	18 (24.7)	2 (11.1)	13 (27.7)	3 (37.5)
Age (years)				
Median (range)	63 (32–76)	63.5 (33–72)	63 (32–76)	57 (39–66)
ECOG PS [n (%)]				
0	40 (54.8)	7 (38.9)	25 (53.2)	8 (100.0)
1	33 (45.2)	11 (61.1)	22 (46.8)	0 (0.0)
Primary tumor location [n (%)]				
G	52 (71.2)	11 (61.1)	35 (74.5)	6 (75.0)
GEJ	21 (28.8)	7 (38.9)	12 (25.5)	2 (25.0)
Histological type [n (%)]				
Signet-ring	14 (19.2)	5 (27.8)	6 (12.8)	3 (37.5)
Non-signet-ring	59 (80.8)	13 (72.2)	41 (87.2)	5 (62.5)
Lauren’s classification [n (%)]				
Intestinal	25 (34.2)	2 (11.1)	22 (46.8)	1 (12.5)
Diffuse	27 (37.0)	6 (33.3)	17 (36.2)	4 (50.0)
Mixed	19 (26.0)	10 (55.6)	6 (12.8)	3 (37.5)
unknown	2 (2.7)	0 (0.0)	2 (4.3)	0 (0.0)
Degree of histological differentiation [n (%)]				
Well	3 (4.1)	1 (5.6)	2 (4.3)	0 (0.0)
Moderately	8 (11.0)	0 (0.0)	8 (17.0)	0 (0.0)
Poorly	50 (68.5)	16 (88.9)	27 (57.4)	7 (87.5)
unknown	12 (16.4)	1 (5.6)	10 (21.3)	1 (12.5)
Clinical T stage [n (%)]				
cT4a	22 (30.1)	18 (100.0)	0 (0.0)	4 (50.0)
cT4b	51 (69.9)	0 (0.0)	47 (100.0)	4 (50.0)
Clinical N stage [n (%)]				
cN0	1 (1.4)	0 (0.0)	0 (0.0)	1 (12.5)
cN1	4 (5.5)	0 (0.0)	4 (8.5)	0 (0.0)
cN2	24 (32.9)	6 (33.3)	14 (29.8)	4 (50.0)
cN3	44 (60.3)	12 (66.7)	29 (61.7)	3 (37.5)
PD-L1 status [n (%)]				
CPS<1	37 (50.7)	10 (55.6)	24 (51.1)	3 (37.5)
CPS≥1	36 (49.3)	8 (44.4)	23 (48.9)	5 (62.5)
CPS<5	55 (75.3)	15 (83.3)	33 (70.2)	7 (87.5)
CPS≥5	18 (24.7)	3 (16.7)	14 (29.8)	1 (12.5)
CPS<10	62 (84.9)	16 (88.9)	39 (83.0)	7 (87.5)
CPS≥10	11 (15.1)	2 (11.1)	8 (17.0)	1 (12.5)
MMR status [n (%)]				
dMMR	7 (9.6)	2 (11.1)	4 (8.5)	1 (12.5)
pMMR	66 (90.7)	16 (88.9)	43 (91.5)	7 (87.5)
HER2 status* [n (%)]				
Positive	8 (11.0)	1 (5.6)	7 (14.9)	0 (0.0)
Negative	65 (89.0)	17 (94.4)	40 (85.1)	8 (100.0)
EBER status [n (%)]				
Positive	2 (2.7)	2 (11.1)	0 (0.0)	0 (0.0)
Negative	35 (48.0)	9 (50.0)	24 (51.1)	2 (25.0)
unknown	36 (49.3)	7 (38.9)	23 (48.9)	6 (75.0)
TMB status (cutoff = 10 muts/Mb) [n (%)]				
high TMB	16 (21.9)	4 (22.2)	9 (19.1)	3 (37.5)
low TMB	41 (56.2)	11 (61.1)	27 (57.5)	3 (37.5)
unknown	16 (21.9)	3 (16.7)	11 (23.4)	2 (25.0)
Treatment regimens [n (%)]				
ICI+CT	24 (32.9)	4 (22.2)	14 (29.8)	6 (75.0)
ICI+CT+AAA	49 (67.1)	14 (77.8)	33 (70.2)	2 (25.0)

AAA, antiangiogenetic agent; CT, Chemotherapy; EBER, EBV-encoded RNA; ECOG PS, Eastern Cooperative Oncology Group performance status; G, Gastric; GEJ, Gastric-esophageal junction; Signet-ring, Signet-ring cell carcinoma; Non-signet-ring, Non-signet-ring cell carcinoma; ICI, Immune checkpoint inhibitor; PD-L1, Programmed cell death-ligand 1; MMR, Mismatch repair; TMB, Tumor mutation burden. *HER2 status was measured using IHC and FISH.

### 3.2 Overall treatment efficacy

**Figure 2:**
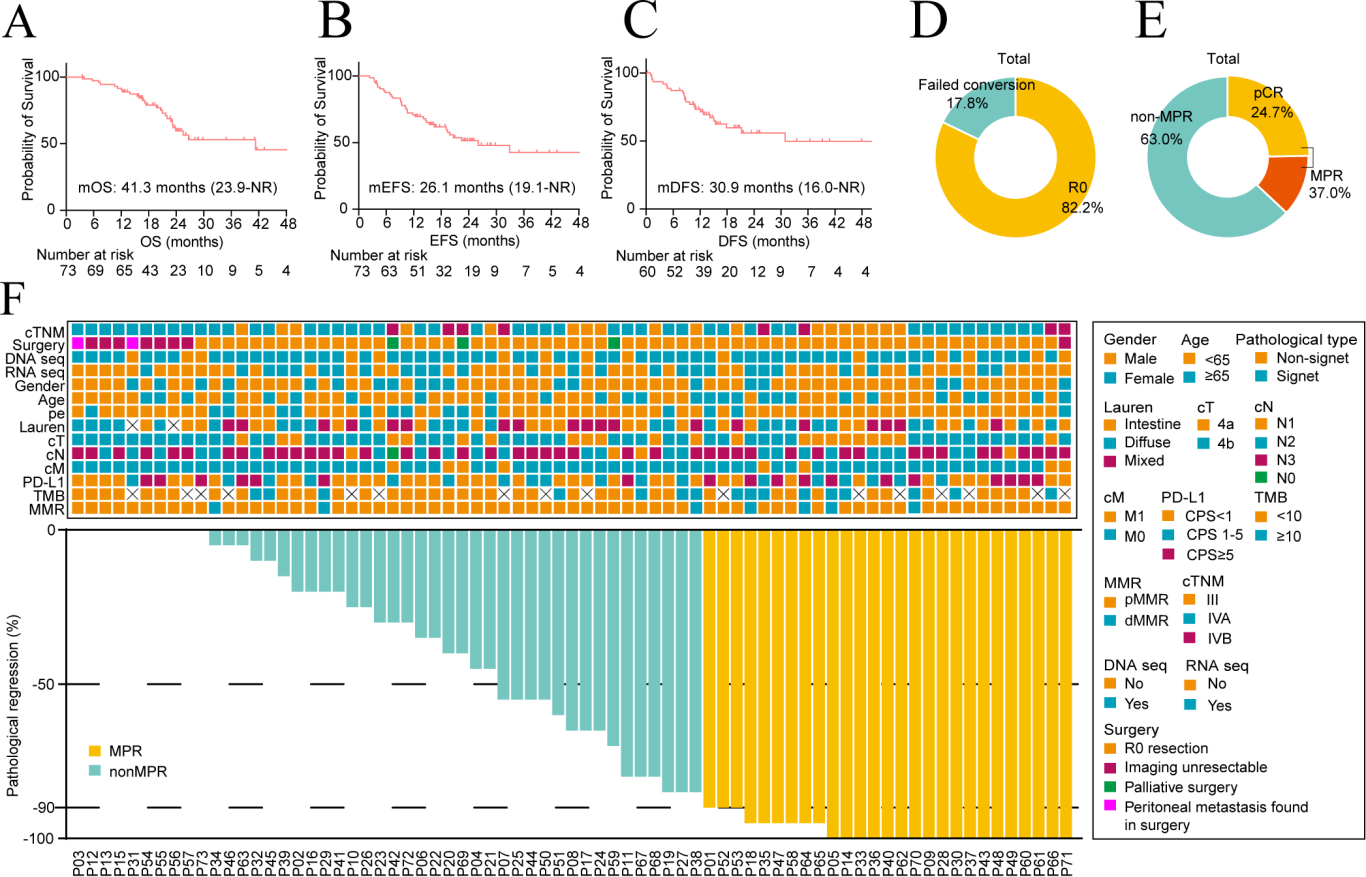
Clinical efficacy of overall patients. **(A)** Kaplan-Meier curves of overall survival (OS) of overall patients. **(B)** Kaplan-Meier curves of event-free survival (EFS) of total patients. **(C)** Kaplan-Meier curves of disease-free survival (DFS) in patients with R0 resection. **(D)** Rates of R0 resection among the overall cohort. **(E)** Rates of pathological complete response (pCR) and major pathological response (MPR) among the overall cohort. **(F)** Waterfall plot visualizing the extent of tumor pathological regression (n = 73). Baseline features are shown on the top panel, while depths of pathological regression are shown at the bottom. CPS, combined positive score; MMR, mismatch repair; PD-L1, programmed death-ligand 1; TMB, tumor mutational burden.

The mOS and mEFS for the 73 patients who received conversion therapy were 41.3 months (95% CI, 23.9–NR) and 26.1 months (95% CI, 19.1–NR), respectively (**Figures 2A, B**). A total of 60 patients (82.2%; 95% CI, 73.2–91.2%) underwent R0 resection (**Figure 2D**), with an mDFS of 30.9 months (95% CI, 16.0–NR) (**Figure 2C**). Conversion treatment failed in 13 patients who did not achieve R0 resection based on clinical or intraoperative evaluation, including three patients who had undergone palliative surgery. One patient underwent palliative total gastrectomy due to peritoneal metastasis, one had a palliative total gastrectomy with bilateral oophorectomy due to adnexal metastasis, one underwent palliative total gastrectomy due to individual physical conditions. Eight patients remained inoperable based on CT examination and did not undergo surgery after conversion therapies following MDT assessment. Two patients underwent exploratory surgery only, as peritoneal metastases were discovered intraoperatively. Among the 63 patients with assessable pathological responses (60 R0 resection and three palliative surgery), the overall pCR and MPR rates were 24.7% (18/73; 95% CI, 14.5–34.8%) and 37.0% (27/73; 95% CI, 25.6–48.3%), respectively (**Figures 2E, F**).

### 3.3 Efficacy analysis according to R0 resection and MPR in overall population

**Figure 3:**
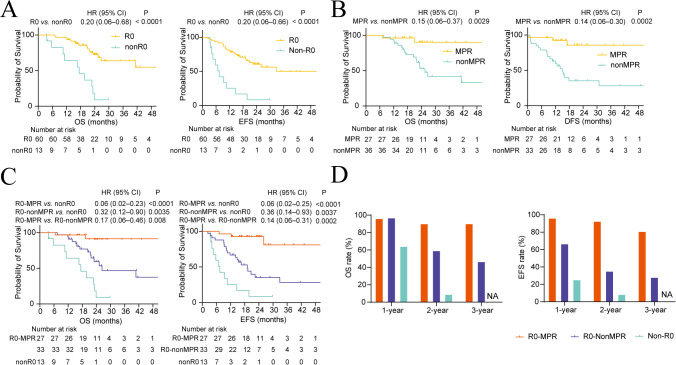
Clinical efficacy stratified by R0 and MPR status. **(A)** Kaplan-Meier curves of OS and EFS stratified according to R0 resection. **(B)** Kaplan-Meier curves of OS and DFS stratified by MPR status. **(C)** Kaplan-Meier curves of OS and EFS of overall patients stratified by R0 and MPR status. Log-rank test was used to assess statistical significance in **(A–C)**. **(D)** 1–3-year OS and EFS rates of overall patients.

Patients who achieved R0 resection had significantly longer OS (HR, 0.20; 95% CI, 0.06–0.68; P < 0.0001) and EFS (HR, 0.20; 95% CI, 0.06–0.66; P < 0.0001) than those who failed to undergo R0 resection (nonR0) (**Figure 3A**). The mEFS and mOS of R0 patients were 32.9 months (95% CI, 22.4–NR) and NR months (95% CI, 26.7–NR), while those for nonR0 patients were only 7.1 months (95% CI, 4.6–NR) and 17.4 months (95% CI, 11.2–NR) (**Figure 3A**).

Among patients undergoing R0 or palliative resection, those who achieved MPR exhibited significantly longer OS (HR, 0.15; 95% CI, 0.06–0.37; P = 0.0029) and DFS (HR, 0.14; 95% CI, 0.06–0.30; P = 0.0002) than those who did not achieve MPR (nonMPR) (**Figure 3B**). The median DFS and OS were not reached in MPR patients, while nonMPR patients had a mDFS of 15.1 months (95% CI, 10.7–NR) and mOS of 25.5 months (95% CI, 22.0–NR) (**Figure 3B**).

R0 resection combined with MPR demonstrated significant OS benefits in both R0–MPR (mOS, NR vs. 17.4 months; HR, 0.06; 95% CI, 0.02–0.23; P < 0.0001) and R0–nonMPR (mOS, 26.7 vs. 17.4 months; HR, 0.32; 95% CI, 0.12–0.90; P = 0.0035) populations compared to nonR0 patients (**Figure 3C**). EFS benefits were also notable in R0–MPR (mEFS, NR vs. 7.4 months; HR, 0.06; 95% CI, 0.02–0.25; P < 0.0001) and R0–nonMPR (mEFS, 19.1 vs. 7.4 months; HR, 0.36; 95% CI, 0.14–0.93; P = 0.0037) relative to nonR0 patients (**Figure 3C**). R0–MPR patients exhibited significant OS and EFS advantages over R0–nonMPR patients (**Figure 3C**). MPR patients had higher survival rates than nonMPR patients at multiple intervals, including 2-year (90.3% vs. 52.8%) and 3-year (90.3% vs. 41.6%) OS rates and 1-year (92.3% vs. 63.1%), 2-year (85.7% vs. 35.3%), and 3-year (85.7% vs. 28.2%) EFS rates (**Figure 3D**, **Supplementary Table S2**).

### 3.4 Efficacy analysis in cIV patients by the combination of R0 resection and MPR

**Figure 4:**
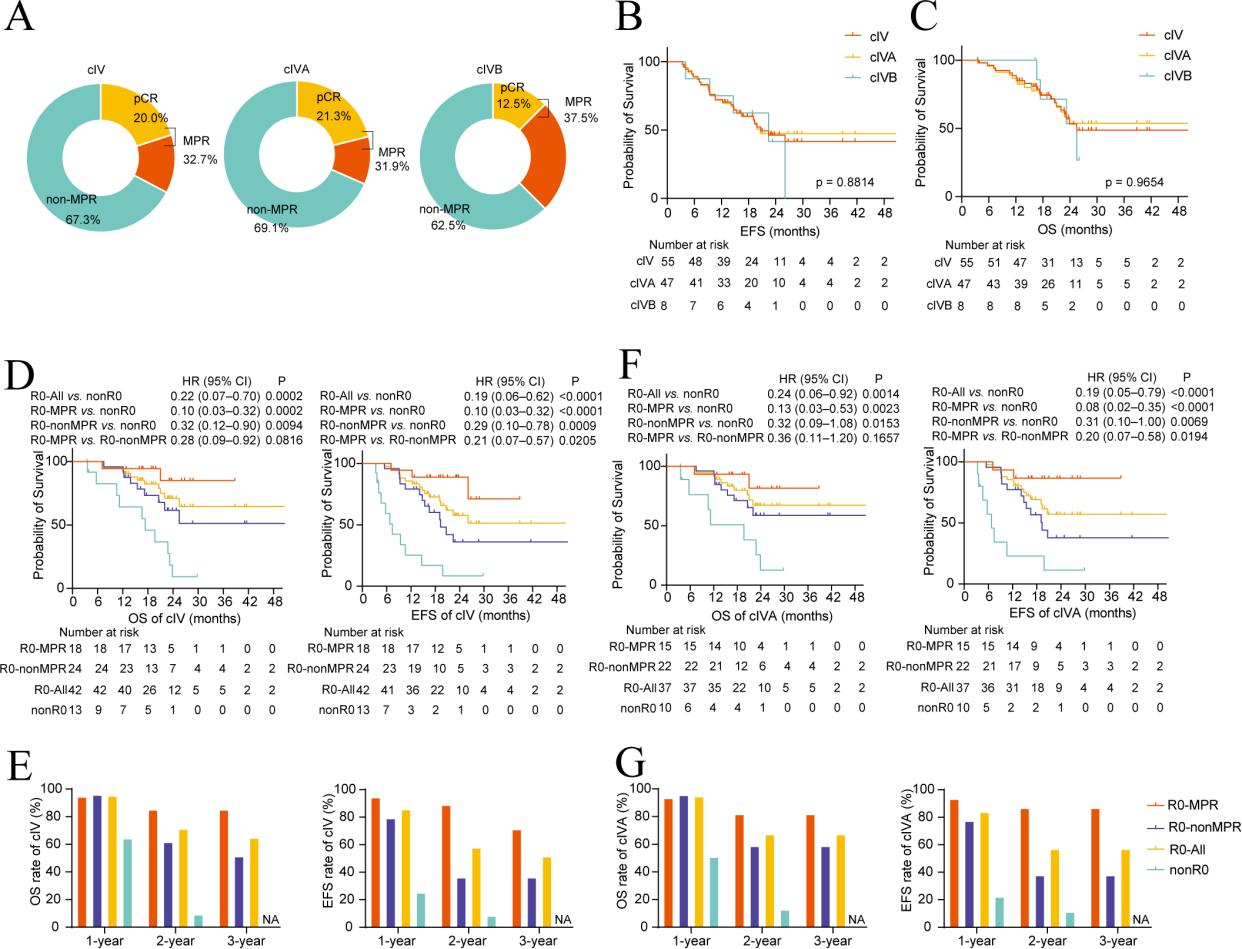
Clinical efficacy analysis in patients with cIV. **(A)** Rates of pathological complete response (pCR) and major pathological response (MPR) in clinical stage cIV, cIVA, and cIVB patients. **(B, C)** Kaplan-Meier curves of event-free survival (EFS) **(B)** and overall survival (OS) **(C)** of cIV, cIVA, and cIVB patients. **(D–G)** Kaplan-Meier curves of OS and EFS of cIV patients **(D)** and cIVA patients **(F)**, stratified by R0 and MPR status, with their 1–3-year OS and EFS rates for cIV **(E)** and IVA **(G)** patients.

Among the 55 patients with cIV, the overall pCR rate was 20.0%, with 21.3% and 12.5% for stages cIVA and cIVB (**Figure 4A**). The MPR rates were 32.7% overall, 31.9% for cIVA, and 37.5% for cIVB (**Figure 4A**). The R0 resection rates were 76.4% overall, 78.7% for cIVA, and 62.5% for cIVB (**Supplementary Figure S1**). Median EFS was 20.7 months and median OS was 25.5 months overall, with mEFS of 20.7 months and mOS not reached for cIVA, and mEFS of 22.4 months and mOS of 25.5 months for cIVB (**Figures 4B, C**). No significant differences in EFS and OS were found among different stages (**Figures 4B, C**). Integrating R0 resection and MPR status offers prognostic insights for cIV patients. Compared to nonR0 patients, R0 patients, including both R0–MPR (mOS, NR vs. 17.4 months; HR, 0.10; 95% CI, 0.03–0.32; P = 0.0002) and R0–nonMPR (mOS, NR vs. 17.4 months; HR, 0.32; 95% CI, 0.12–0.90; P = 0.0094), showed better OS. Similarly, EFS benefits were significant in both R0–MPR (mEFS, NR vs. 7.1 months; HR, 0.10; 95% CI, 0.03–0.32; P < 0.0001) and R0–nonMPR (mEFS, 19.3 months vs. 7.1 months; HR, 0.29; 95% CI, 0.10–0.78; P = 0.0009) compared to nonR0 patients. Additionally, R0–MPR patients had significant EFS benefits and a beneficial trend in OS compared to R0–nonMPR patients (**Figure 4D**). Patients with R0–MPR, R0–nonMPR, and nonR0 had 1-year OS rates of 94.4%, 95.8%, and 64.2%; 2-year OS rates of 85.0%, 61.6%, and 9.2%; and 3-year OS rates of 85.0%, 51.3%, and approximately 0%, respectively. These EFS rate trends mirrored those in OS (**Figure 4E**, **Supplementary Table S2**).

In the cIVA group, R0–MPR patients experienced significant OS and EFS benefits compared to nonR0 patients, while R0–nonMPR patients had significant EFS benefits and a trend toward OS benefit over nonR0 patients. Additionally, R0–MPR patients exhibited significant EFS and OS benefit trends compared to R0–nonMPR cIVA patients (**Figure 4F**). The cIVA patients demonstrated similar OS and EFS trends as cIV patients (**Figure 4G**, **Supplementary Table S2**).

### 3.5 Clinical efficacy of patients with different clinicopathological characteristics

**Figure 5:**
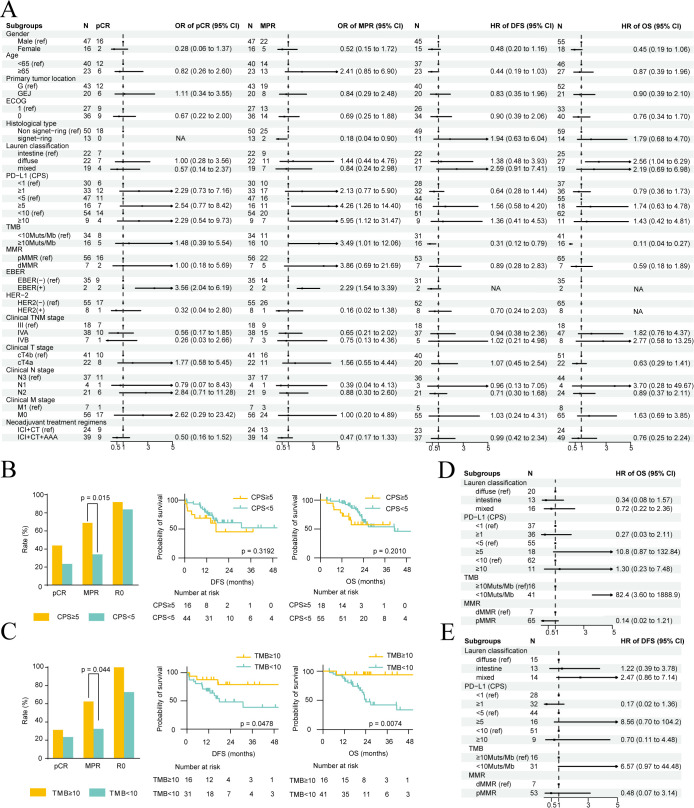
Clinical efficacy according to different clinicopathological characteristics. **(A)** Pathological complete response (pCR), major pathological response (MPR) rate, disease-free survival (DFS) and overall survival (OS) rates among different subgroups. Odds ratios (ORs) were calculated by Chi-square test, while hazard ratios (HRs) were obtained by univariable Cox analysis. **(B, C)** Comparison of rates of pCR, MPR, and R0 resection (left), and DFS and OS (right) among patients with different PD-L1 CPS **(B)** or TMB **(C)** statuses. **(D, E)** Multivariable Cox analyses for OS **(D)** and EFS **(E)** AAA, antiangiogenic agent. CT, chemotherapy. ECOG, Eastern Cooperative Oncology Group. G, gastric. GEJ, gastroesophageal junction. ICI, immune checkpoint inhibitor. MMR, mismatch repair. PD-L1, programmed death-ligand 1. TMB, tumor mutational burden.

Univariable subgroup analysis revealed that EBER positivity was the only factor associated with pCR (OR = 3.56, 95% CI: 2.04–6.19) (**Figure 5A**). Elevated MPR was significantly associated with non-signet-ring cell carcinoma, PD-L1 CPS ≥5, PD-L1 CPS ≥10, and TMB-H (**Figures 5A–C**). TMB-H was also associated with longer DFS and OS, whereas Lauren’s intestinal type was associated with longer OS (**Figures 5A, C**). MMR status was not significantly associated with pCR, MPR, OS, and DFS. Age, gender, and location did not significantly impact OS and DFS (**Figure 5A**, **Supplementary Figure S2**). In multivariable Cox models including variables with P < 0.05 in univariable analyses and biologically relevant immunotherapy covariates (PD-L1, MMR), TMB-H emerged as the sole factor independently associated with OS and DFS after adjusting for potential confounders (**Figures 5D, E**). The adjusted VIFs for variables in these models were all below 2.0 (**Supplementary Table S3**), indicating that multicollinearity did not unduly influence the estimates. The independent prognostic value of TMB-H was further validated through sensitivity analyses. The significant association between TMB-H and survival outcomes remained consistent after sequentially excluding MMR status, Lauren classification, or PD-L1 expression from the multivariable models, underscoring the robustness of this finding (**Supplementary Figure S3**). It should be noted that the subgroup analyses for certain biomarkers, particularly HER2-positive (n = 8), dMMR/MSI-H (n = 7), and EBER-positive (n = 2) populations, are limited by small sample sizes.

### 3.6 Safety profile and postoperative complications

The incidence of grade ≥3 TRAEs was 16.4% (95% CI, 7.8–25.5%), including myelosuppression, anorexia, nausea, fatigue, and hypokalemia, with no new toxicities or treatment-related deaths (**Supplementary Table S4**). The median time from the last treatment to surgery was 17.5 days (quantile, 14.3–23.8), and the median hospital stay was 12.0 days (quantile, 10.0–13.8). Grade ≥3 postoperative complications, including anastomotic fistula, duodenal stump fistula, and pleural effusion, occurred in 3.2% (95% CI, 0.0–6.7%) of patients (**Supplementary Table S5**). One patient (P61) died from COVID-19 on postoperative day 10.

### 3.7 Association between KMT2D mutations and efficacy

**Figure 6:**
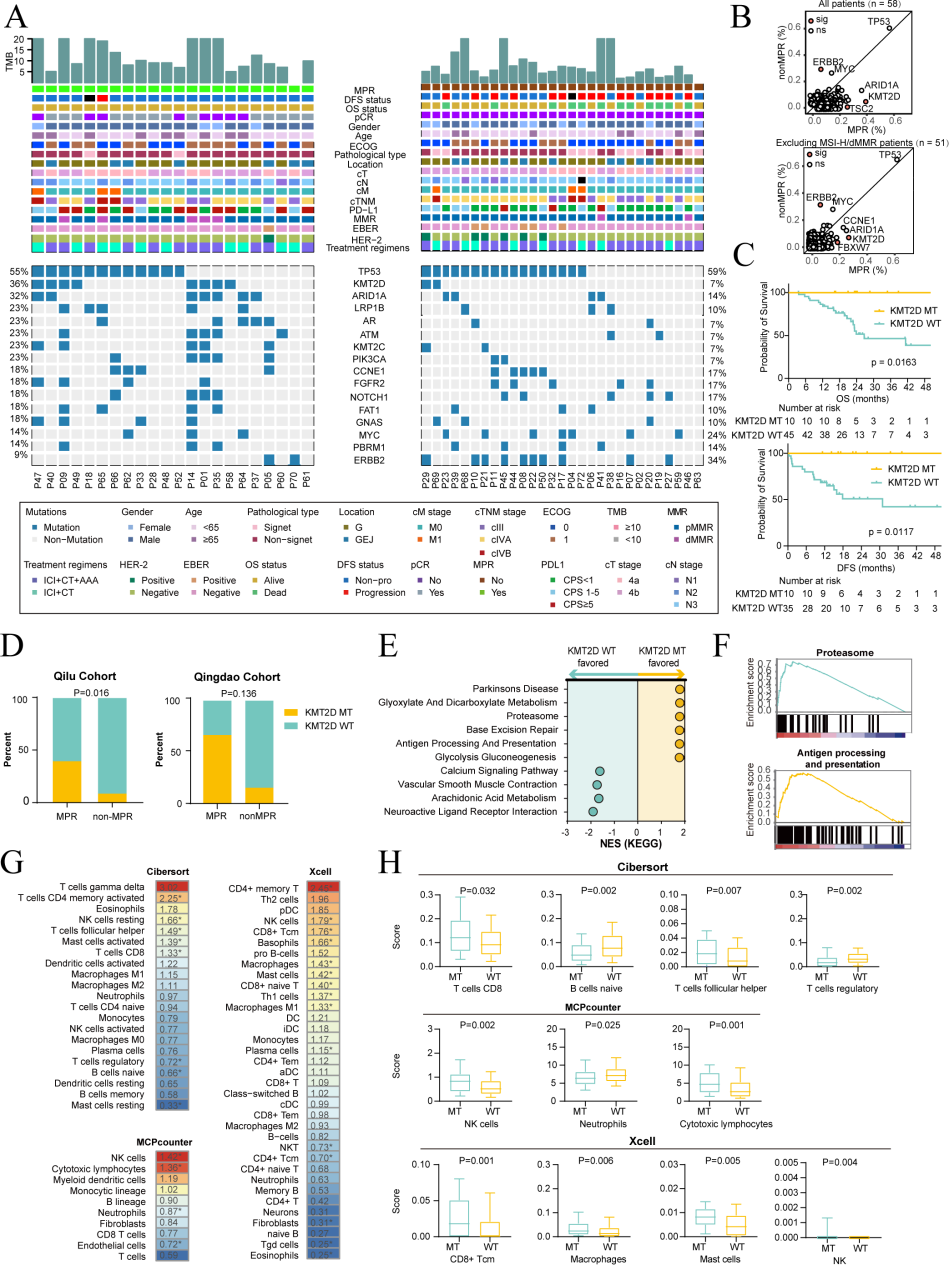
Association of KMT2D mutation with major pathological response and immune landscape. **(A)** Mutational landscape of major pathological response (MPR; n = 22)/nonMPR (n = 29) patients. TMB and baseline features are shown in the upper panel, while the top 20 genes with the highest mutation frequency are in the lower panel. **(B)** Analysis of the association between gene mutations and pathological response. Upper panel: Differences in gene mutation frequencies between the MPR and nonMPR groups among the 58 patients who underwent genomic profiling. Lower panel: Sensitivity analysis performed after excluding all MSI-H/dMMR patients to mitigate potential confounding effects of dMMR status on the results. Each dot represents a gene, and orange dots indicate significance. **(C)** Kaplan-Meier curves comparing overall survival (OS) and disease-free survival (DFS) stratified by KMT2D mutation status. Log-rank test was used to evaluate survival differences. **(D)** Prevalence of KMT2D mutations in MPR and nonMPR populations in Qilu Cohort (left) and Qingdao Cohort (right). **(E)** Comparison of pathway enrichments between KMT2D mutation and wild-type patients by Gene Set Enrichment Analysis (GSEA), with the normalized enrichment score (NES) indicating pathway activation levels. **(F)** GSEA enrichment analysis of “Proteasome” and “Antigen processing and presentation” pathways, comparing KMT2D mutation and wild-type patients. **(G)** Fold changes of immune cell levels between KMT2D mutation and wild-type patients by Cibersort, Xcell, and MCPcounter methods. The number represents fold changes and * denotes significance. **(H)** Comparison of levels of specific immune cells between KMT2D mutation and wild-type patients. **(E–H)** used data from the TCGA stomach cancer cohort. AAA, antiangiogenic agent; CT, chemotherapy; ECOG, Eastern Cooperative Oncology Group; G, gastric; GEJ, gastroesophageal junction; ICI, immune checkpoint inhibitor; MMR, mismatch repair; PD-L1, programmed death-ligand 1; TMB, tumor mutational burden; non-pro, no progression; TCGA, the cancer genome atlas.

Genomic analysis identified TP53, KMT2D, and ARID1A as the most commonly altered genes (**Figure 6A**). In MPR patients, pathways such as “non-homologous end joining” and “ubiquitin-mediated proteolysis” were frequently altered, whereas “adherent junction” was prevalent in nonMPR patients (**Supplementary Figure S4A**). A significant association was found between KMT2D mutation and MPR, while ERBB2 alteration was significantly linked to nonMPR, even excluding MSI-H or HER2-positive patients (**Figure 6B**, **Supplementary Figure S4B**). KMT2D mutation was not significantly associated with TMB, PD-L1 expression, or cTNM staging (**Supplementary Figure S4C**) but was significantly correlated with improved OS (P = 0.0163), DFS (P = 0.0117), and MPR (P = 0.016) (**Figures 6C, D**). The Qingdao Cohort validated the link between KMT2D mutations and MPR, with a higher mutation rate in patients with MPR (66.7% vs. 15.4%, P = 0.136) (**Figure 6D**). Analysis of the TCGA GC database showed that KMT2D mutant tumors were active in “proteasome” and “antigen processing and presentation” pathways (**Figures 6E, F**, **Supplementary Figures S4D–E**), with increased infiltration of CD8+ T cells, NK cells, and cytotoxic lymphocytes (**Figures 6G, H**). Conversely, ERBB2 alteration was significantly associated with a lower MPR rate (P = 0.025) (**Supplementary Figure S4F**) but did not impact OS, DFS (**Supplementary Figure S4G**). In the Qingdao Cohort, none of the patients with ERBB2 mutations or amplifications achieved MPR (**Supplementary Figure S4F**).

### 3.8 Association of the P450 signature with tumor immune microenvironments and responses

**Figure 7:**
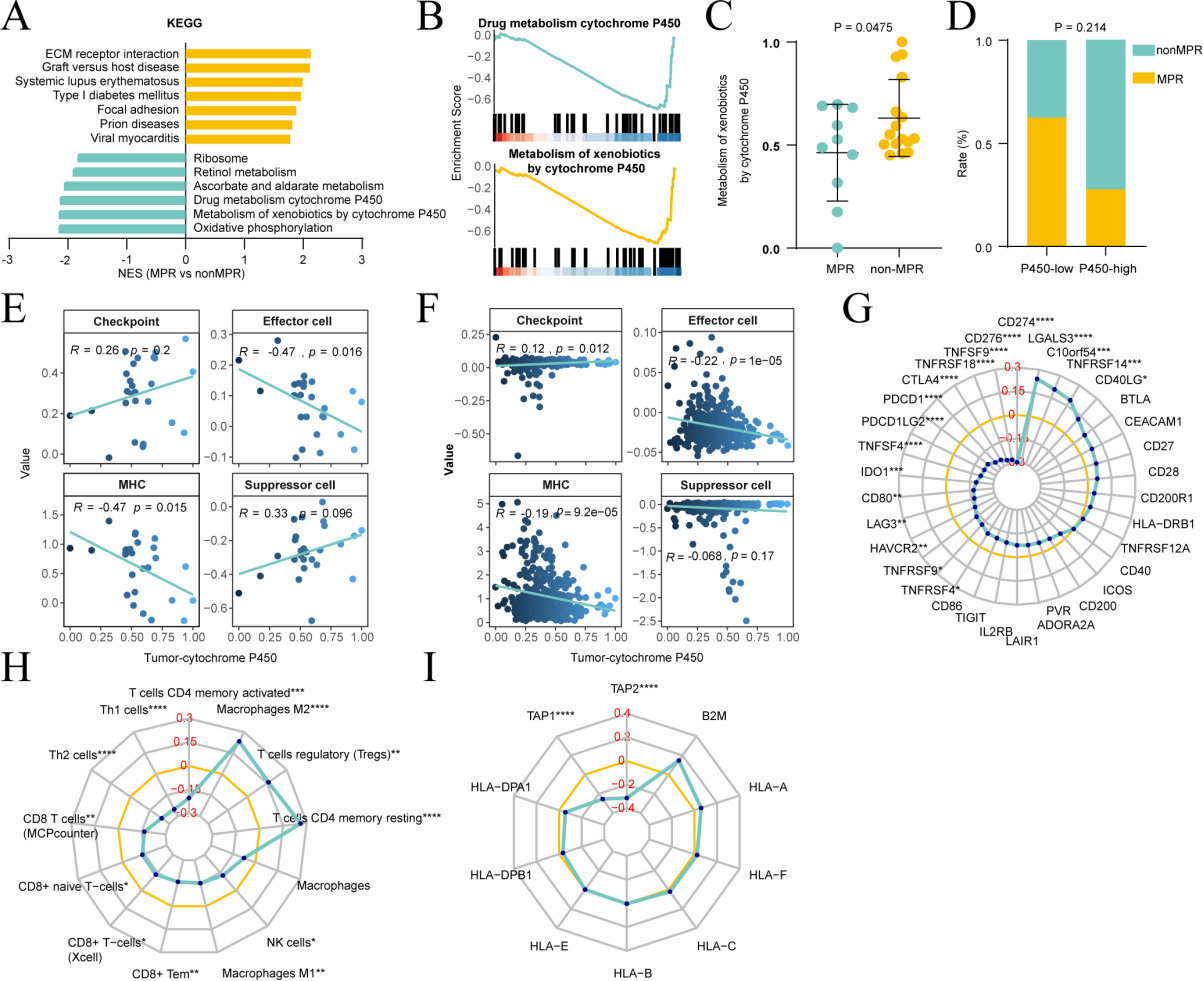
Correlation of major pathological response (MPR) with tumor cytochrome P450 activity. **(A)** Comparison of pathway enrichments between MPR and nonMPR patients by Gene Set Enrichment Analysis (GSEA), with the normalized enrichment score (NES) indicating pathway activation levels. **(B)** GSEA enrichment analysis focusing on the “Drug metabolism cytochrome P450” and “Metabolism of xenobiotics by cytochrome P450” pathways comparing MPR and nonMPR patients. **(C)** Comparison of the “Metabolism of xenobiotics by cytochrome P450” signature scores between MPR and nonMPR populations. **(D)** Comparison of MPR rates between P450-high versus -low populations. **(E, F)** Correlation between tumor-cytochrome P450 signatures and immunophenotypic scores of “Checkpoint”, “Effector cell”, “MHC”, and “Suppressor cell” in the Qilu Cohort **(E)** and TCGA Cohort **(F)**. **(G–I)** Spider plots depicting the correlation coefficients between tumor-cytochrome P450 signature and specific immune parameters in “Checkpoint” **(G)**, “Effector/Suppressor cell” **(H)**, and “MHC” **(I)**. The yellow circle denotes the threshold for positive/negative correlation, and * represents significance. *P < 0.05; **P < 0.01; ***P < 0.001; ****P < 0.0001.

RNA sequencing unveiled distinct pathway landscapes; the MPR group was enriched in immune-related pathways, while the nonMPR group was enriched in P450-related pathways (**Figures 7A, B**). Consequently, patients with MPR had lower P450 signature scores than those without MPR (**Figure 7C**), and the proportion of nonMPR patients increased in those with high P450 scores (**Figure 7D**). Differentially expressed genes between the MPR/nonMPR groups were also significantly enriched in two P450-related pathways, with several genes overexpressed in nonMPR patients (**Supplementary Figures S5A, B**).

The Immunophenogram revealed that tumor-P450 negatively correlated with effector cells and MHC, and positively associated with checkpoints and suppressing cell markers in both the investigated cohort and TCGA-STAD cohort (**Figures 7E, F**, **Supplementary Figure S6**). P450-high tumors exhibited upregulated checkpoint molecules, including CTLA4, PDCD1, CD274, and LAG3 (**Figure 7G**). The P450 signature was negatively correlated with CD8+ T cells, NK cells, and M1 macrophages, and positively correlated with M2 macrophages and Treg cells (**Figure 7H**). Furthermore, MHC molecules TAP1 and TAP2 negatively correlated with the P450 signature (**Figure 7I**). To synthesize these multi-omics findings and provide clearer mechanistic insight, a schematic diagram has been added as **Supplementary Figure S7**.

## 4 Discussion

As mentioned above, studies on conversion immunotherapy for initially unresectable or oligometastatic GC are limited. Furthermore, ongoing phase III RCTs on neoadjuvant immunotherapy included only 1.1% to 4% of patients with stage cIVA or cIVB (11–13). Given the considerable proportion of this patient group within the overall GC demographics, the effectiveness of conversion therapy is highly relevant to survival outcomes (2, 5, 6). However, previous studies on stage cIV GC conversion therapy predominantly employed chemotherapy, with limited efficacy (5, 6). It is therefore important to examine the efficacy of immunotherapy for conversion and its determinants in this patient population.

Regarding the pathological assessment of treatment response, it is important to clarify the criteria employed in this study. While various TRG systems (e.g., Becker, Mandard, Japanese Classification) are indeed used in gastric cancer neoadjuvant therapy, we standardized our definitions by aligning them with specific TRG categories from these systems: for instance, Becker TRG1a, Mandard TRG1, and JCGC TRG3 were considered equivalent to pCR. This approach was taken to harmonize data across referenced studies. Furthermore, we note that recent pivotal phase III trials evaluating neoadjuvant immunotherapy in gastric cancer [such as KEYNOTE-585 (11), MATTERHORN (12), and DRAGON-IV (13)] have adopted pCR as endpoints. Therefore, the use of pCR and MPR as key efficacy metrics in our analysis is not only methodologically sound but also aligns with contemporary clinical research standards, facilitating meaningful cross-trial comparisons.

In this study, 75% of patients had cIVA (cT4bN+M0) or cIVB, and the remaining cIII patients were deemed initially unresectable by the MDT. The application of ICI-based conversion therapy and subsequent surgery yielded an R0 resection rate of 82.2%, a pCR rate of 24.7%, an MPR rate of 37.0%, an mEFS of 26.1 months, and an mOS of 41.3 months. Patients with stage cIVA achieved a nearly 80% R0 resection rate and a 53.8% 3-year OS rate, while those with cIVB reached an R0 resection rate of 62.5%, surpassing previous reports for chemotherapy alone (24). It is noteworthy that patients with cIVB achieved a mEFS of 17.0 months and a mOS of 25.5 months, surpassing the approximately 15-month mOS reported in earlier RCTs assessing first-line ICIs with chemotherapy for advanced GC (25).

Compared to perioperative immunotherapy trials like KEYNOTE-585 and MATTERHORN, which predominantly included patients with stage cII–III disease (>95%), this study had only 25% stage cIII patients, yet achieved equivalent R0 resection rates (82.2%)—comparable to KEYNOTE-585 (80%) (11), although lower than MATTERHORN (91%) (12) and DRAGON-IV (98.7%) (13). This high R0 resection rate, reflecting successful conversion treatment, significantly enhanced survival. Notably, the pCR rate was 24.7% in our study, which was higher than that reported in KEYNOTE-585 (12.9%) (11), MATTERHORN (19%) (12), and DRAGON-IV (18.3%) (13). For initially unresectable stage cIV patients, OS was notably longer in R0 patients compared to nonR0, with an 80% reduction in the risk of death and recurrence and an 8-fold increase in 2-year EFS/OS rates. The 3-year EFS and OS rates for R0 patients rose to 51.4% and 64.6%, respectively, exceeding the efficacy of chemotherapy alone (24, 26). Notably, patients achieving MPR following R0 resection exhibited even greater benefits, with a 90% reduction in the risk of death or recurrence and significantly higher 3-year OS (85.0% vs. near 0%) and EFS (71.1% vs. near 0%) compared to nonR0 patients.

In stage cIV GC patients with R0 resection, OS did not differ significantly between patients with and without MPR, while stage cIV nonMPR patients had significantly better survival than nonR0 ones. Therefore, radical surgery after successful conversion appears crucial for patients with advanced GC. Those who did not achieve MPR after radical surgery may still benefit from R0 resection, as subsequent treatments can offset limited short-term efficacy (27). This real-world evidence highlights the necessity for ICI-based conversion therapy followed by R0 surgery to extend survival in patients with locally advanced unresectable or oligometastatic GC. These findings help inform existing research for this patient group and may support the rationale for large RCTs to evaluate the comprehensive strategy of ICI-based conversion therapy followed by radical surgery, with the goal of improving survival outcomes.

PD-L1, TMB, EBER, and dMMR/MSI are recognized as efficacy predictors for immunotherapy in advanced GC (28), but their predictive value in neoadjuvant/conversion immunotherapy remains unclear. Our study showed that increased PD-L1 expression significantly improved pCR and MPR rates without extending EFS and OS, consistent with the KEYNOTE-585 study (11). This suggests that PD-L1’s predictive value in perioperative or conversion immunotherapy requires further verification. Conversely, the TMB-H subgroup not only had higher MPR rates but also longer OS and DFS than the TMB-L subgroup, indicating that TMB may have a better prognostic predictive value than PD-L1. TMB quantifies somatic mutations and approximates neoantigen load, and is associated with immunogenicity and response to immunotherapy (29, 30). While TMB’s predictive value has been established in palliative settings for advanced disease (31), its role in conversion therapy has been underexplored. Our study demonstrates that TMB−H was independently associated with benefit in unresectable GC undergoing immunotherapy−based conversion. Furthermore, trends toward improved prognosis were observed in EBER-positive and dMMR/MSI-H subgroups, while HER2 positivity showed no significant association with efficacy outcomes. However, due to the limited number of patients in these subgroups, these analyses are inherently underpowered, and the results should be interpreted with caution. These findings suggest that the applicability of these markers in neoadjuvant or conversion treatment settings remains unclear and warrants further investigation in larger, prospective studies.

Genomic analysis identified a link between KMT2D mutations and enhanced efficacy, specifically associated with increased antitumor immune cell infiltration, aligning with previous findings in colorectal adenocarcinoma (32). Mechanistically, immune-related pathways such as “proteasome” and “antigen processing and presentation” were significantly enriched in patients with KMT2D mutations, likely explaining the increased immune cell infiltration and a more immunologically active TME (33). This generates the hypothesis that inhibiting wild-type KMT2D function might convert immunologically “cold tumors” into “hot tumors” and improve immunotherapy responsiveness in GC. Small-molecule inhibitors targeting KMT2D have been developed (34), offering potential strategies to enhance the sensitivity of KMT2D wild-type GC to immunotherapy.

Transcriptomic analyses indicated that nonMPR patients’ genes were notably enriched in the P450 pathway, suggesting a connection with immunotherapy resistance in GC. The P450 enzyme system, known for detoxification (35), has an unclear role in affecting the tumor immune microenvironment and resistance to immunotherapy, with sparse research in animal models (36). Our data provide clinical evidence linking a high P450 transcriptional signature to a suppressed antitumor immune phenotype, characterized by upregulation of multiple immune checkpoint molecules (e.g., PD-1, CTLA-4, LAG-3), increased infiltration of immunosuppressive cells (e.g., M2 macrophages, Tregs), and impaired antigen presentation machinery. Targeting CYP19A1 within the P450 system has shown promise in boosting CD8+ T cell anti-tumor activity and improving anti-PD-1 therapy outcomes (37). Collectively, these findings from our study and others generate the compelling hypothesis that the P450 pathway may act as a metabolic regulator of immune evasion in GC. These results imply that the P450 system could potentially facilitate immunotherapy resistance by altering antitumor immune responses, suggesting that combining P450 system inhibition with ICIs might offer a new approach to overcome immunotherapy resistance in GC.

Consistent with its exploratory aim, this study has several inherent limitations that define the scope of its conclusions. It was a real-world, single-arm investigation lacking a contemporaneous control group. Given the retrospective design, limited sample size, and the inherent exploratory nature of this study, we caution against overgeneralization of the results. Second, the external validation cohort’s small size and baseline/regimen imbalances may also limit interpretability, weaken statistical power, and introduce selection bias. Therefore, the biomarker analysis primarily serves for “hypothesis generation” rather than “definitive confirmation”, and subsequent validation in larger, prospective, and randomized controlled trials is necessary. To translate these findings into clinical practice, we have initiated a multicenter prospective study to validate the predictive value of KMT2D mutations and the P450 signature. In parallel, functional studies using KMT2D-edited models and patient-derived organoids are underway to elucidate their mechanistic roles in shaping the tumor immune microenvironment.

## 5 Conclusions

ICI-based conversion therapy shows promising survival benefits and an acceptable safety profile in patients with unresectable locally advanced or oligometastatic GC, supporting its potential as a treatment strategy. Furthermore, our exploratory multi-omics analyses shed light on potential drug resistance mechanisms and uncovered candidate biomarkers associated with treatment efficacy, which warrant further investigation.

## Data Availability

The datasets presented in this study can be found in online repositories. The names of the repository/repositories and accession number(s) can be found below: https://ngdc.cncb.ac.cn/omix/, OMIX012153 https://ngdc.cncb.ac.cn/omix/, OMIX012154.

## References

[1] Sung H Ferlay J Siegel RL Laversanne M Soerjomataram I Jemal A . Global cancer statistics 2020: GLOBOCAN estimates of incidence and mortality worldwide for 36 cancers in 185 countries. CA Cancer J Clin. (2021) 71:209–49. doi: 10.3322/caac.21660, PMID: 33538338

[2] Zeng H Ran X An L Zheng R Zhang S Ji JS . Disparities in stage at diagnosis for five common cancers in China: a multicentre, hospital-based, observational study. Lancet Public Health. (2021) 6:e877–877e887. doi: 10.1016/S2468-2667(21)00157-2, PMID: 34838194

[3] Lu Z Zhang X Liu W Liu T Hu B Li W . A multicenter, randomized trial comparing efficacy and safety of paclitaxel/capecitabine and cisplatin/capecitabine in advanced gastric cancer. Gastric Cancer. (2018) 21:782–91. doi: 10.1007/s10120-018-0809-y, PMID: 29488121 PMC6097104

[4] Al-Batran SE Homann N Pauligk C Goetze TO Meiler J Kasper S . Perioperative chemotherapy with fluorouracil plus leucovorin, oxaliplatin, and docetaxel versus fluorouracil or capecitabine plus cisplatin and epirubicin for locally advanced, resectable gastric or gastro-oesophageal junction adenocarcinoma (FLOT4): a randomised, phase 2/3 trial. Lancet. (2019) 393:1948–57. doi: 10.1016/S0140-6736(18)32557-1, PMID: 30982686

[5] Kinoshita J Yamaguchi T Moriyama H Fushida S . Current status of conversion surgery for stage IV gastric cancer. Surg Today. (2021) 51:1736–54. doi: 10.1007/s00595-020-02222-0, PMID: 33486610

[6] Al-Batran SE Homann N Pauligk C Illerhaus G Martens UM Stoehlmacher J . Effect of neoadjuvant chemotherapy followed by surgical resection on survival in patients with limited metastatic gastric or gastroesophageal junction cancer: the AIO-FLOT3 trial. JAMA Oncol. (2017) 3:1237–44. doi: 10.1001/jamaoncol.2017.0515, PMID: 28448662 PMC5824287

[7] Zhang Z Huang J Xu Y Luo H . Chemoimmunotherapy for esophageal squamous cell carcinoma—Summary and discussion of recent clinical trials. MedComm – Future Med. (2023) 2:e56. doi: 10.1002/mef2.56

[8] Zheng Y Huang J Xu Y Luo H . Current progress in chimeric antigen receptor T-cell therapy for Malignant tumors. MedComm – Future Med. (2024) 3:e79. doi: 10.1002/mef2.79

[9] Bagchi S Yuan R Engleman EG . Immune checkpoint inhibitors for the treatment of cancer: clinical impact and mechanisms of response and resistance. Annu Rev Pathol. (2021) 16:223–49. doi: 10.1146/annurev-pathol-042020-042741, PMID: 33197221

[10] Kang YK Chen LT Ryu MH Oh DY Oh SC Chung HC . Nivolumab plus chemotherapy versus placebo plus chemotherapy in patients with HER2-negative, untreated, unresectable advanced or recurrent gastric or gastro-oesophageal junction cancer (ATTRACTION-4): a randomised, multicentre, double-blind, placebo-controlled, phase 3 trial. Lancet Oncol. (2022) 23:234–47. doi: 10.1016/S1470-2045(21)00692-6, PMID: 35030335

[11] Shitara K Rha SY Wyrwicz LS Oshima T Karaseva N Osipov M . Neoadjuvant and adjuvant pembrolizumab plus chemotherapy in locally advanced gastric or gastro-oesophageal cancer (KEYNOTE-585): an interim analysis of the multicentre, double-blind, randomised phase 3 study. Lancet Oncol. (2024) 25:212–24. doi: 10.1016/S1470-2045(23)00541-7, PMID: 38134948

[12] Janjigian YY Al-Batran SE Wainberg ZA Muro K Molena D Van Cutsem E . Perioperative Durvalumab in Gastric and Gastroesophageal Junction Cancer. *N Engl J Med*. (2025) 393:217–30. doi: 10.1056/NEJMoa2503701, PMID: 40454643

[13] Li C Tian Y Zheng Y Yuan F Shi Z Yang L . Pathologic Response of Phase III Study: Perioperative Camrelizumab Plus Rivoceranib and Chemotherapy Versus Chemotherapy for Locally Advanced Gastric Cancer (DRAGON IV/CAP 05). *J Clin Oncol*. (2025) 43:464–74. doi: 10.1200/JCO.24.00795, PMID: 39383487 PMC11776878

[14] Smyth EC Nilsson M Grabsch HI van Grieken NC Lordick F . Gastric cancer. Lancet. (2020) 396:635–48. doi: 10.1016/S0140-6736(20)31288-5, PMID: 32861308

[15] Sexton RE Al Hallak MN Diab M Azmi AS . Gastric cancer: a comprehensive review of current and future treatment strategies. Cancer Metastasis Rev. (2020) 39:1179–203. doi: 10.1007/s10555-020-09925-3, PMID: 32894370 PMC7680370

[16] Lorenzen S Götze TO Thuss-Patience P Biebl M Homann N Schenk M . Perioperative Atezolizumab Plus Fluorouracil, Leucovorin, Oxaliplatin, and Docetaxel for Resectable Esophagogastric Cancer: Interim Results From the Randomized, Multicenter, Phase II/III DANTE/IKF-s633 Trial. J Clin Oncol. (2024) 42:410–20. doi: 10.1200/JCO.23.00975, PMID: 37963317

[17] Li H Deng J Ge S Zang F Zhang L Ren P . Phase II study of perioperative toripalimab in combination with FLOT in patients with locally advanced resectable gastric/gastroesophageal junction (GEJ) adenocarcinoma. J Clin Oncol. (2026) 39:4050–4050. doi: 10.1200/JCO.2021.39.15_suppl.4050

[18] Li N Li Z Fu Q Zhang B Zhang J Wan X . Phase II study of sintilimab combined with FLOT regimen for neoadjuvant treatment of gastric or gastroesophageal junction (GEJ) adenocarcinoma. *J Clin Oncol*. (2026) 39:216–216. doi: 10.1200/JCO.2021.39.3_suppl.216

[19] Dindo D Demartines N Clavien PA . Classification of surgical complications: a new proposal with evaluation in a cohort of 6336 patients and results of a survey. Ann Surg. (2004) 240:205–13. doi: 10.1097/01.sla.0000133083.54934.ae, PMID: 15273542 PMC1360123

[20] Aran D Hu Z Butte AJ . xCell: digitally portraying the tissue cellular heterogeneity landscape. Genome Biol. (2017) 18:220. doi: 10.1186/s13059-017-1349-1, PMID: 29141660 PMC5688663

[21] Newman AM Steen CB Liu CL Gentles AJ Chaudhuri AA Scherer F . Determining cell type abundance and expression from bulk tissues with digital cytometry. Nat Biotechnol. (2019) 37:773–82. doi: 10.1038/s41587-019-0114-2, PMID: 31061481 PMC6610714

[22] Becht E Giraldo NA Lacroix L Buttard B Elarouci N Petitprez F . Estimating the population abundance of tissue-infiltrating immune and stromal cell populations using gene expression. Genome Biol. (2016) 17:218. doi: 10.1186/s13059-016-1070-5, PMID: 27765066 PMC5073889

[23] Charoentong P Finotello F Angelova M Mayer C Efremova M Rieder D . Pan-cancer immunogenomic analyses reveal genotype-immunophenotype relationships and predictors of response to checkpoint blockade. Cell Rep. (2017) 18:248–62. doi: 10.1016/j.celrep.2016.12.019, PMID: 28052254

[24] Sato Y Ohnuma H Nobuoka T Hirakawa M Sagawa T Fujikawa K . Conversion therapy for inoperable advanced gastric cancer patients by docetaxel, cisplatin, and S-1 (DCS) chemotherapy: a multi-institutional retrospective study. Gastric Cancer. (2017) 20:517–26. doi: 10.1007/s10120-016-0633-1, PMID: 27553665

[25] Rha SY Oh DY Yañez P Bai Y Ryu MH Lee J . Pembrolizumab plus chemotherapy versus placebo plus chemotherapy for HER2-negative advanced gastric cancer (KEYNOTE-859): a multicentre, randomised, double-blind, phase 3 trial. Lancet Oncol. (2023) 24:1181–95. doi: 10.1016/S1470-2045(23)00515-6, PMID: 37875143

[26] Yamaguchi K Yoshida K Tanahashi T Takahashi T Matsuhashi N Tanaka Y . The long-term survival of stage IV gastric cancer patients with conversion therapy. Gastric Cancer. (2018) 21:315–23. doi: 10.1007/s10120-017-0738-1, PMID: 28616743 PMC5846815

[27] Li S Xu Q Dai X Zhang X Huang M Huang K . Neoadjuvant therapy with immune checkpoint inhibitors in gastric cancer: A systematic review and meta-analysis. Ann Surg Oncol. (2023) 30:3594–602. doi: 10.1245/s10434-023-13143-w, PMID: 36795255

[28] Xie T Liu Y Zhang Z Zhang X Gong J Qi C . Positive status of epstein-barr virus as a biomarker for gastric cancer immunotherapy: A prospective observational study. J Immunother. (2020) 43:139–44. doi: 10.1097/CJI.0000000000000316, PMID: 32134806 PMC7144749

[29] Jardim DL Goodman A de Melo Gagliato D Kurzrock R . The challenges of tumor mutational burden as an immunotherapy biomarker. Cancer Cell. (2021) 39:154–73. doi: 10.1016/j.ccell.2020.10.001, PMID: 33125859 PMC7878292

[30] Samstein RM Lee CH Shoushtari AN Hellmann MD Shen R Janjigian YY . Tumor mutational load predicts survival after immunotherapy across multiple cancer types. Nat Genet. (2019) 51:202–6. doi: 10.1038/s41588-018-0312-8, PMID: 30643254 PMC6365097

[31] Lee KW Van Cutsem E Bang YJ Fuchs CS Kudaba I Garrido M . Association of tumor mutational burden with efficacy of pembrolizumab ± Chemotherapy as first-line therapy for gastric cancer in the phase III KEYNOTE-062 study. Clin Cancer Res. (2022) 28:3489–98. doi: 10.1158/1078-0432.CCR-22-0121, PMID: 35657979

[32] Liu C Jin Y Zhang H Yan J Guo Y Bao X . Effects of KMT2D mutation and its exon 39 mutation on the immune microenvironment and drug sensitivity in colorectal adenocarcinoma. Heliyon. (2023) 9:e13629. doi: 10.1016/j.heliyon.2023.e13629, PMID: 36846668 PMC9950945

[33] Wang G Chow RD Zhu L Bai Z Ye L Zhang F . CRISPR-GEMM pooled mutagenic screening identifies KMT2D as a major modulator of immune checkpoint blockade. Cancer Discov. (2020) 10:1912–33. doi: 10.1158/2159-8290.CD-19-1448, PMID: 32887696 PMC7710536

[34] Yu Q Liao Z Liu D Xie W Liu Z Liao G . Small molecule inhibitors of the prostate cancer target KMT2D. Biochem Biophys Res Commun. (2020) 533:540–7. doi: 10.1016/j.bbrc.2020.09.004, PMID: 32988590

[35] Stipp MC Acco A . Involvement of cytochrome P450 enzymes in inflammation and cancer: a review. Cancer Chemother Pharmacol. (2021) 87:295–309. doi: 10.1007/s00280-020-04181-2, PMID: 33112969

[36] Chen C Yang Y Guo Y He J Chen Z Qiu S . CYP1B1 inhibits ferroptosis and induces anti-PD-1 resistance by degrading ACSL4 in colorectal cancer. Cell Death Dis. (2023) 14:271. doi: 10.1038/s41419-023-05803-2, PMID: 37059712 PMC10104818

[37] Liu L Mo M Chen X Chao D Zhang Y Chen X . Targeting inhibition of prognosis-related lipid metabolism genes including CYP19A1 enhances immunotherapeutic response in colon cancer. J Exp Clin Cancer Res. (2023) 42:85. doi: 10.1186/s13046-023-02647-8, PMID: 37055842 PMC10100168

