# Supplementary material for: Survival and multi-omics analysis of immunotherapy-based conversion treatment for initially unresectable gastric cancer
Source: Front Immunol. 2026 Jan 13;16:1753749. doi: 10.3389/fimmu.2025.1753749 (PMC12835316; doi:10.3389/fimmu.2025.1753749)
Supplement: Supplementary file 1 [file DataSheet1.docx]

Supplementary Material

# Supplementary Figures

**
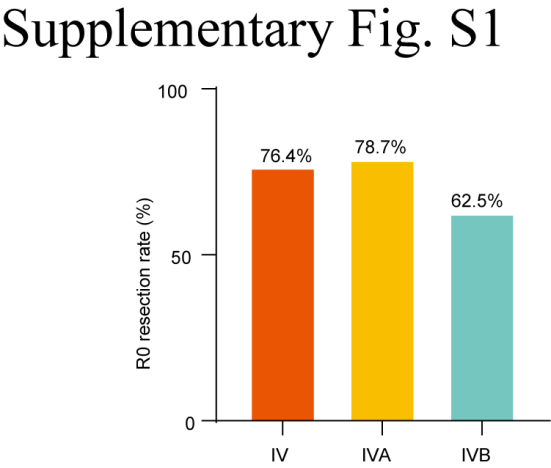
**

**Supplementary Figure 1. R0 resection rate of clinical IV, IVA and IVB patients.**

**
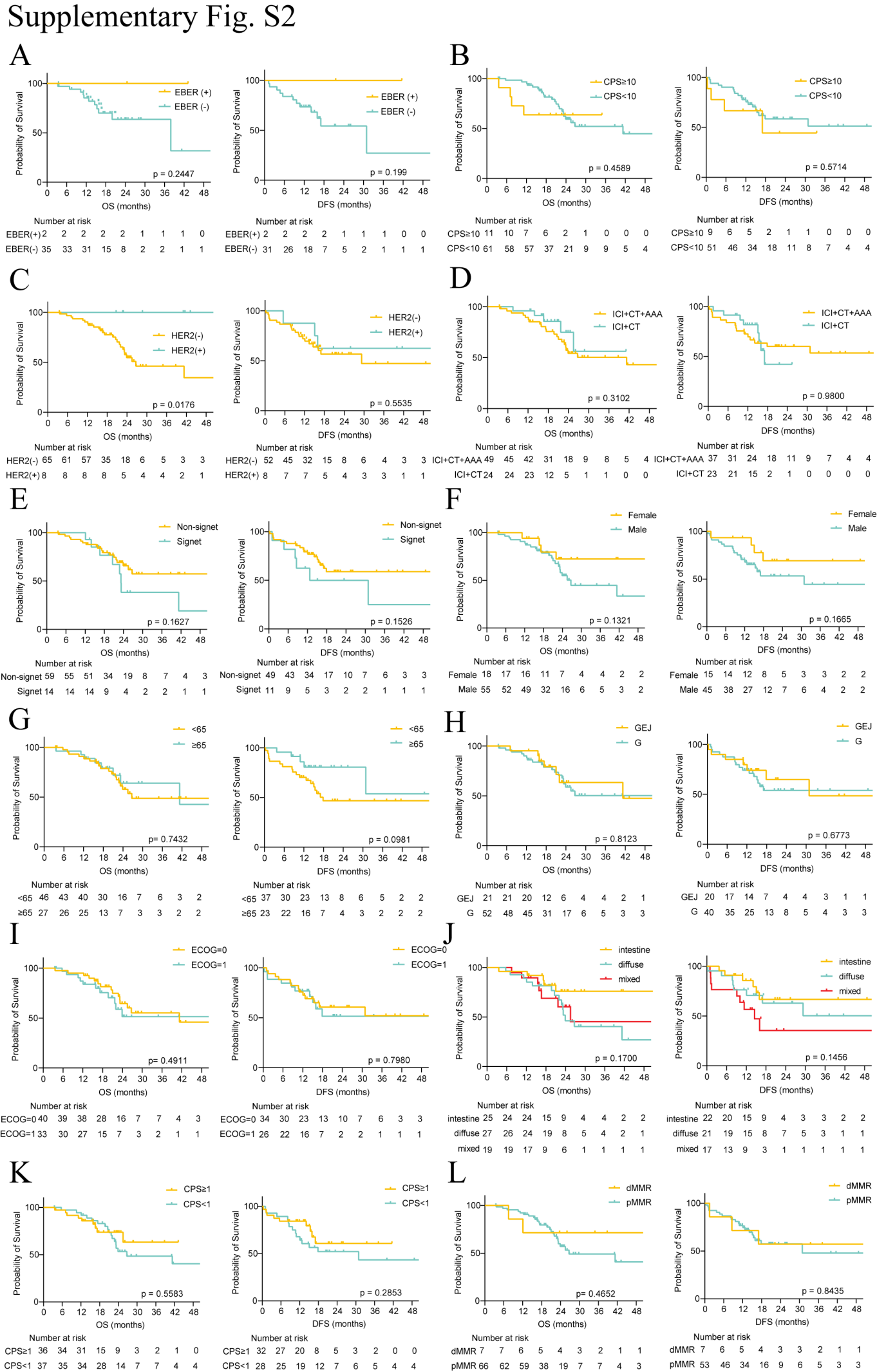
**

**Supplementary Figure 2. Subgroup analysis for overall survival and disease-free survival.** Kaplan-Meier curves of OS and DFS among patients with different EBER expression (A), PD-L1 CPS (10) (B), HER2 expression (C), treatment regimens (D), pathological types (E), gender (F), age (G), location (H), ECOG (I), Lauren’s classification (J), PD-L1 CPS (K) and MMR status (L). Log-rank test was used to assess statistical significance. AAA, antiangiogenic agent; CPS, combined positive score; CT, chemotherapy; ECOG, Eastern Cooperative Oncology Group; G, gastric; GEJ, gastroesophageal junction; ICI, immune checkpoint inhibitor; MMR, mismatch repair.

**
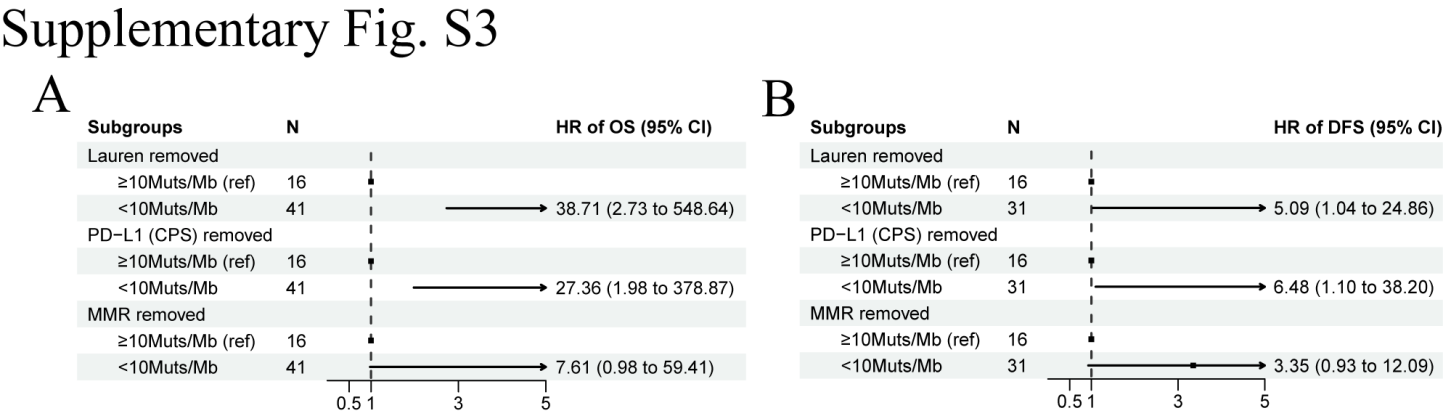
**

**Supplementary Figure 3. Sensitivity analysis of multivariate COX analyses for overall survival (A) and disease-free survival (B).**

**
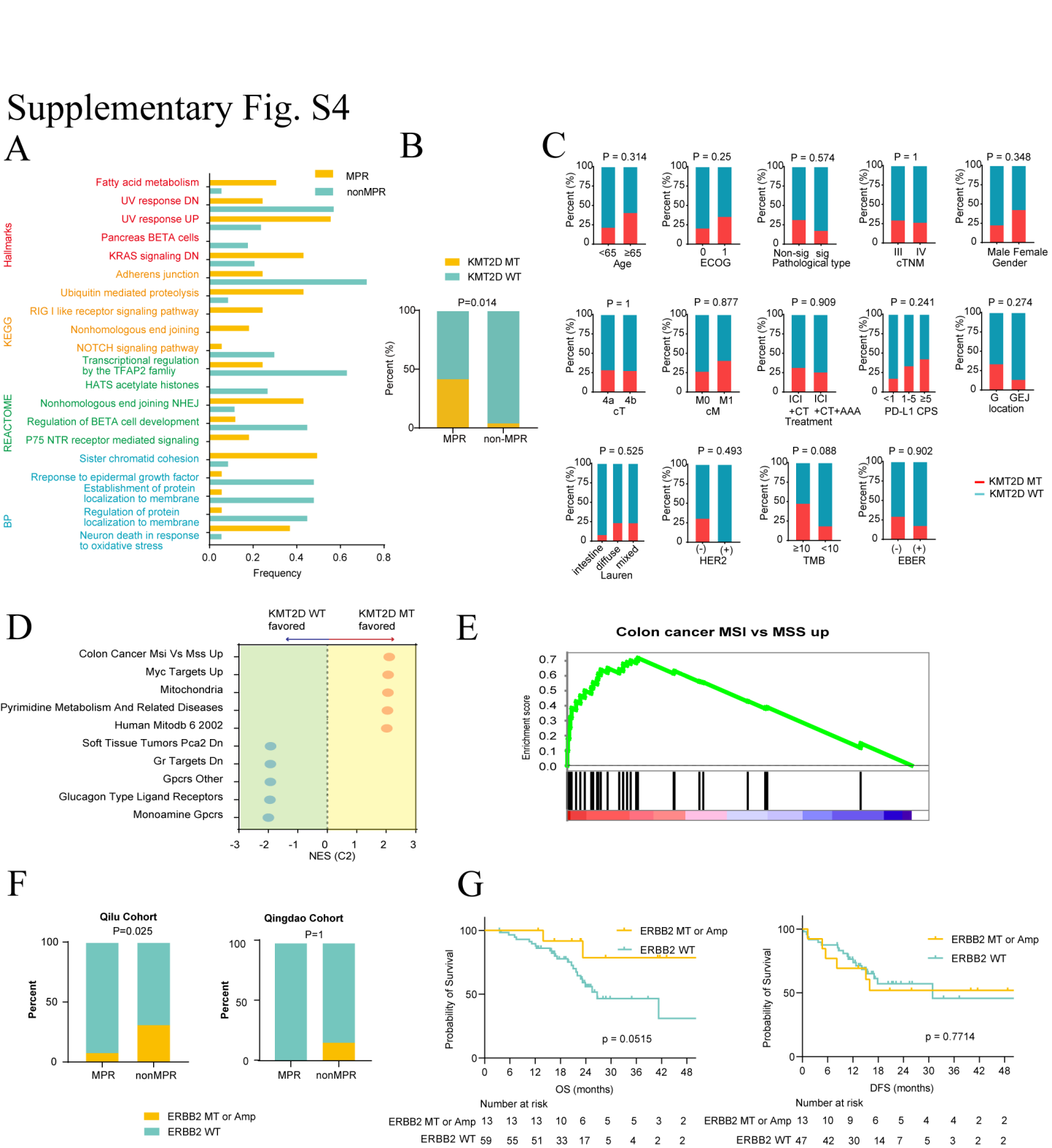
**

**Supplementary Figure 4. Comprehensive analysis of genetic variants and pathway enrichments in response to treatment.** (A) Functional pathway enrichment by mutated genes in major pathological response (MPR) and nonMPR patients. (B) Prevalence of KMT2D mutations in MPR and nonMPR populations in Qilu Cohort without HER2-positive patients. (C) Frequencies of *KMT2D* mutations across different subgroups. (D) Comparison of C2 pathway enrichments between *KMT2D* mutation and wild-type patients, with the normalized enrichment score (NES) indicating pathway activation levels. (E) Gene Set Enrichment Analysis (GSEA) enrichment analysis of “Colon cancer MSI vs MSS up” pathway, comparing *KMT2D* mutation and wild-type patients. (F) Prevalence of *ERBB2* alternations in MPR and nonMPR populations in Qilu Cohort (left) and Qingdao Cohort (right). (G) Kaplan-Meier curves comparing overall survival (OS) and disease-free survival (DFS) stratified by *ERBB2* alternation status. Log-rank test was used to evaluate survival differences. AAA, antiangiogenic agent; CPS, combined positive score; CT, chemotherapy; ECOG, Eastern Cooperative Oncology Group; G, gastric; GEJ, gastroesophageal junction; ICI, immune checkpoint inhibitor; PD-L1, programmed death-ligand 1; TMB, tumor mutational burden.

**
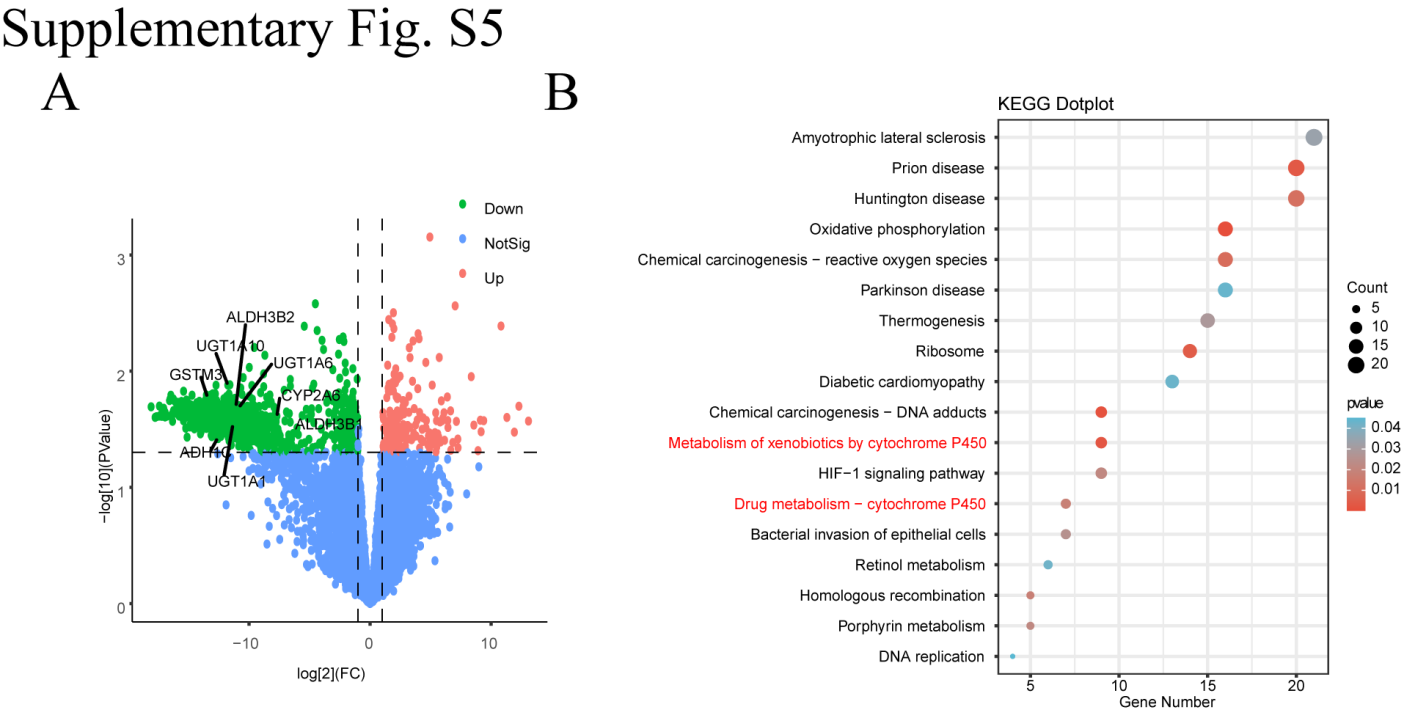
**

**Supplementary Figure 5. Differential gene enrichment analysis between major pathological response (MPR) and nonMPR patients.** (A) Volcano plot showing differentially expressed genes between MPR and nonMPR patients. Color dots denote genes that passed the p-value and fold change thresholds (Red favored MPR group, green favored nonMPR group). (B) Bubble map of differential gene enrichment analysis in KEGG pathway between MPR and nonMPR patients.

**
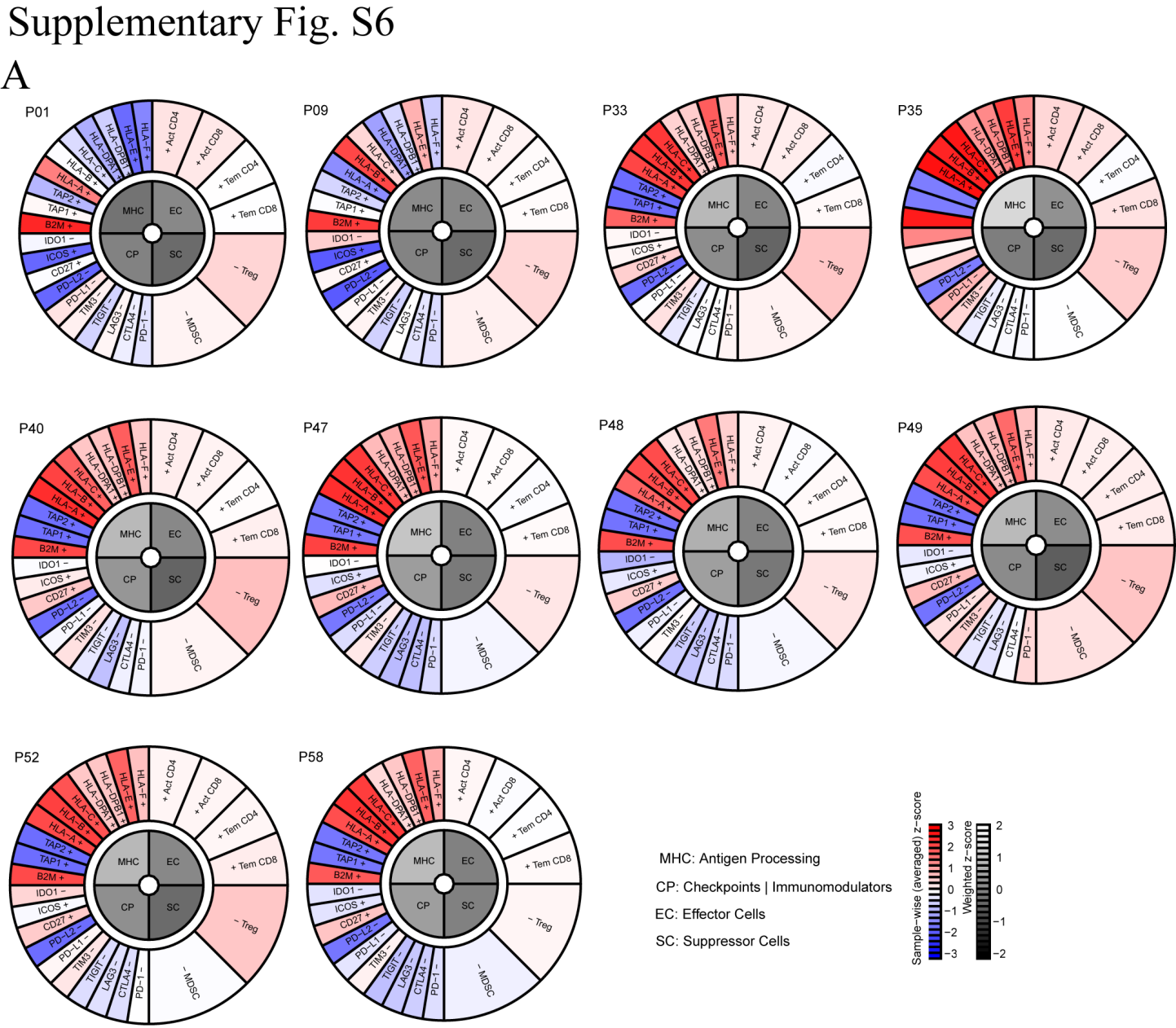
**

**
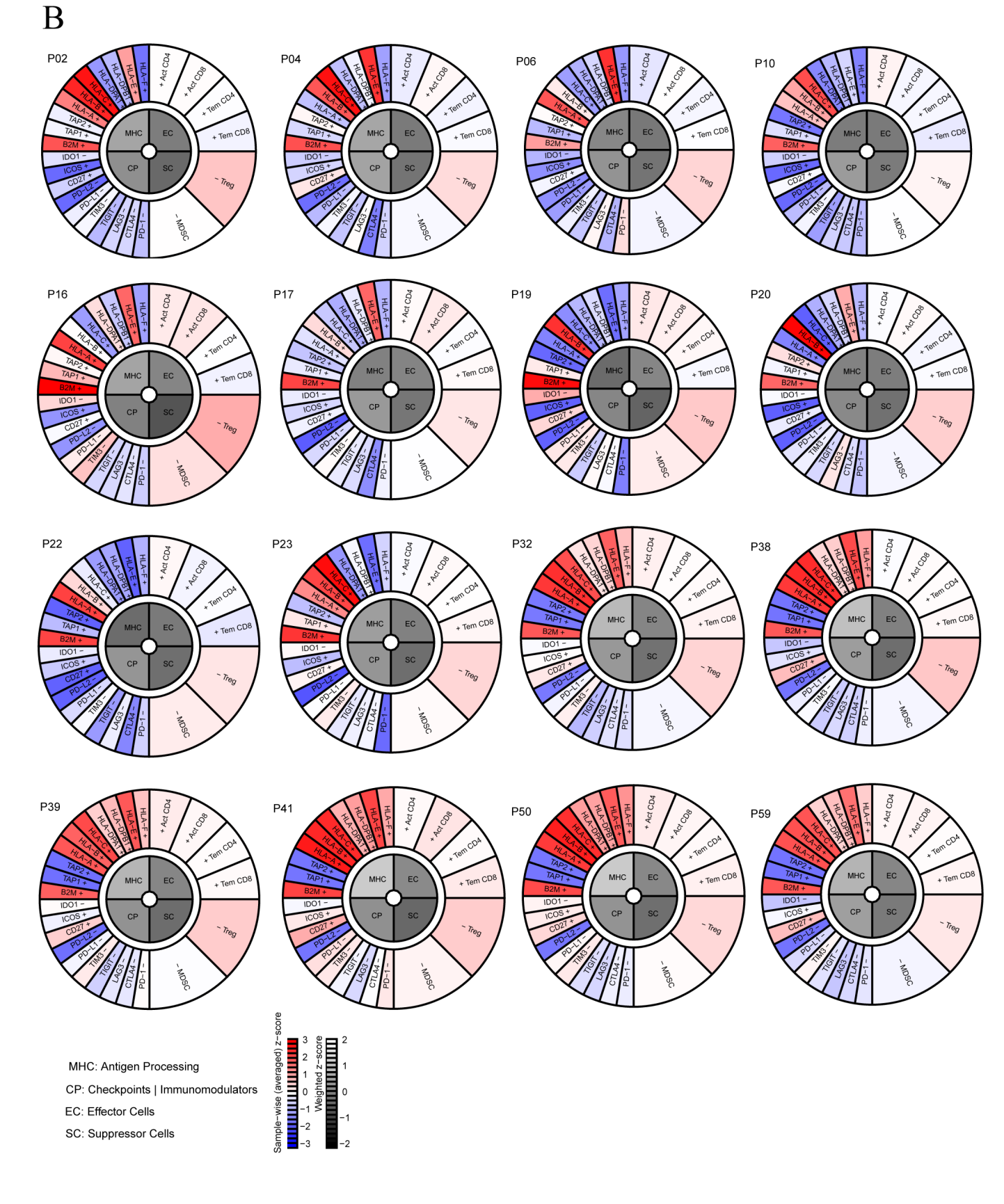
**

**Supplementary Figure 6. Immunophenotypes of patients with major pathological response (MPR) or nonMPR.** The major determinants are involved in four categories: MHC molecules (MHC), immunomodulators (CP), effector cells (EC), and suppressor cells (SC). n = 10 (MPR) and 16 (nonMPR).


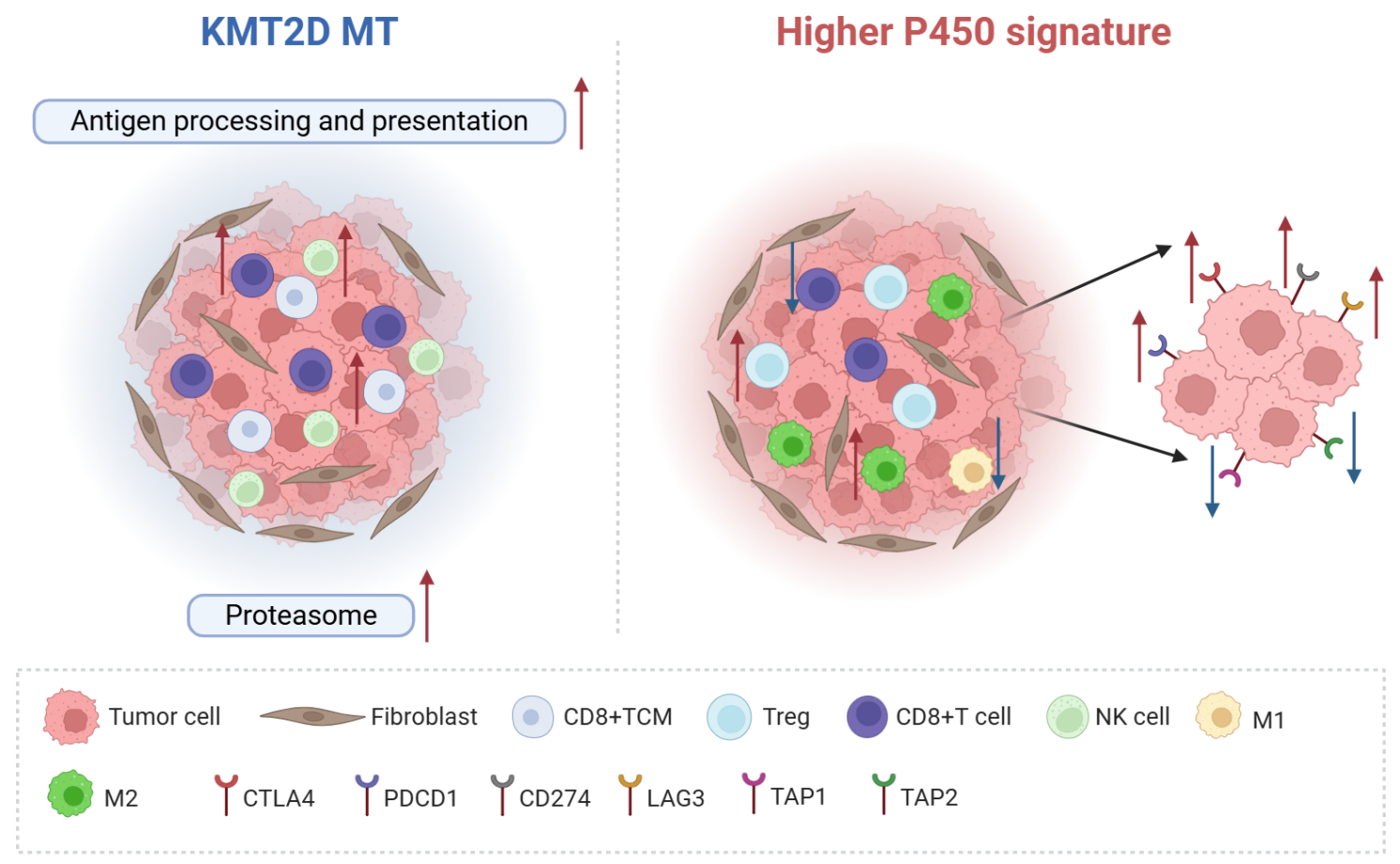


**Supplementary Figure 7. Schematic diagram summarizing proposed mechanisms underlying KMT2D mutation-associated immune activation and P450 signature-linked immunotherapy resistance in gastric cancer.**

# Supplementary Tables

**Supplementary Table 1. Summary of treatment.**

| **Variables** | | **Number (%)** |
| --- | --- | --- |
| Treatment cycles | |  |
|  | 2 | 24 (32.9) |
|  | 3 | 15 (20.5) |
|  | 4 | 4 (5.5) |
|  | 5 | 12 (16.4) |
|  | 6 | 9 (12.3) |
|  | 7 | 7 (9.6) |
|  | 8 | 2 (2.8) |
| Treatment regimens | |  |
|  | ICI+Apatinib+S-1 | 11 (15.0) |
|  | ICI+Apatinib+SOX | 38 (52.1) |
|  | ICI+SOX+Trastuzumab | 1 (1.4) |
|  | ICI+SOX | 23 (31.5) |

**Supplementary Table 2. OS, DFS and EFS rate in different subgroups.**

| **Variables** | | **1-year rate** | |  | **2-year rate** | |  | **3-year rate** | |
| --- | --- | --- | --- | --- | --- | --- | --- | --- | --- |
|  |  | Rate (%) | 95% CI |  | Rate (%) | 95% CI |  | Rate (%) | 95% CI |
| OS | |  |  |  |  |  |  |  |  |
|  | Overall population | 91.6 | 85.3-98.3 |  | 59.6 | 47.6-74.5 |  | 52.9 | 40.0-69.9 |
|  | cIII | 100.0 | 100.0-100.0 |  | 77.4 | 57.8-100.0 |  | 64.5 | 40.6-100.0 |
|  | cIV | 88.7 | 80.6-97.6 |  | 53.2 | 39.3-71.9 |  | 48.8 | 34.5-69.0 |
|  | cIVA | 86.7 | 77.4-97.2 |  | 53.8 | 39.0-74.2 |  | 53.8 | 39.0-74.2 |
|  | cIVB | 100.0 | 100.0-100.0 |  | 53.6 | 25.7-100.0 |  | NA | NA |
|  | cIV R0-All | 95.2 | 89.0-100.0 |  | 71.1 | 57.0-87.0 |  | 64.6 | 48.4-86.3 |
|  | cIV nonR0 | 64.2 | 41.3-99.6 |  | 9.2 | 1.4-59.3 |  | NA | NA |
|  | cIV R0-MPR | 94.4 | 84.4-100.0 |  | 85.0 | 67.2-100.0 |  | 85.0 | 67.2-100.0 |
|  | cIV R0-nonMPR | 95.8 | 88.2-100.0 |  | 61.6 | 43.4-87.4 |  | 51.3 | 31.1-84.6 |
|  | cIVA R0-All | 94.6 | 87.6-100.0 |  | 67.2 | 51.9-87.0 |  | 67.2 | 51.9-87.0 |
|  | cIVA nonR0 | 50.8 | 25.7-100.0 |  | 12.7 | 2.0-79.1 |  | NA | NA |
|  | cIVA R0-MPR | 93.3 | 81.5-100.0 |  | 81.7 | 60.8-100.0 |  | 81.7 | 60.8-100.0 |
|  | cIVA R0-nonMPR | 95.5 | 87.1-100.0 |  | 58.7 | 39.9-86.2 |  | 58.7 | 39.9-86.2 |
|  | MPR | 96.3 | 89.4-100.0 |  | 90.3 | 78.0-100.0 |  | 90.3 | 78.0-100.0 |
|  | nonMPR | 94.4 | 87.3-100.0 |  | 52.8 | 37.0-75.3 |  | 41.6 | 25.5-67.7 |
| DFS | |  |  |  |  |  |  |  |  |
|  | Overall population | 76.2 | 66.0-87.9 |  | 57.2 | 44.6-73.3 |  | 50.8 | 36.2-71.4 |
|  | cIII | 66.2 | 47.4-92.4 |  | 66.2 | 47.4-92.4 |  | 53.0 | 30.5-91.9 |
|  | cIV | 80.5 | 69.2-93.6 |  | 53.0 | 37.8-74.2 |  | 53.0 | 37.8-74.2 |
|  | cIVA | 77.7 | 65.2-92.7 |  | 55.7 | 40.3-77.2 |  | 55.7 | 40.3-77.2 |
|  | cIVB | 100.0 | 100.0-100.0 |  | NA | NA |  | NA | NA |
|  | MPR | 92.3 | 82.6-100.0 |  | 85.7 | 71.4-100.0 |  | 85.7 | 71.4-100.0 |
|  | nonMPR | 63.1 | 48.5-82.1 |  | 35.3 | 21.0-59.1 |  | 28.2 | 14.3-55.6 |
| EFS | |  |  |  |  |  |  |  |  |
|  | Overall population | 70.9 | 61.1-82.2 |  | 51.4 | 40.2-65.7 |  | 42.6 | 29.6-61.3 |
|  | cIV R0-All | 85.7 | 75.8-97.0 |  | 57.8 | 43.0-77.7 |  | 51.4 | 35.3-74.8 |
|  | cIV nonR0 | 25.4 | 9.6-67.3 |  | 8.5 | 1.3-55.1 |  | NA | NA |
|  | cIV R0-MPR | 94.4 | 84.4-100.0 |  | 88.9 | 75.5-100.0 |  | 71.1 | 44.5-100.0 |
|  | cIV R0-nonMPR | 79.2 | 64.5-97.2 |  | 36.1 | 19.6-66.4 |  | 36.1 | 19.6-66.4 |
|  | cIVA R0-All | 83.8 | 72.7-96.5 |  | 56.9 | 41.6-77.7 |  | 56.9 | 41.6-77.7 |
|  | cIVA nonR0 | 22.9 | 6.8-76.8 |  | 11.4 | 1.8-72.0 |  | NA | NA |
|  | cIVA R0-MPR | 93.3 | 81.5-100.0 |  | 86.7 | 71.1-100.0 |  | 86.7 | 71.1-100.0 |
|  | cIVA R0-nonMPR | 77.3 | 61.6-96.9 |  | 37.8 | 20.8-68.7 |  | 37.8 | 20.8-68.7 |

**Supplementary Table 3. Summary of VIF in multivariate COX analysis.**

| **Variables** | | **GVIF** | **Adjusted VIF** |
| --- | --- | --- | --- |
| Overall survival | |  |  |
|  | PD-L1 | 1.12 | 1.06 |
|  | MMR | 1.74 | 1.32 |
|  | TMB | 1.81 | 1.35 |
|  | Lauren’s classification | 1.09 | 1.02 |
| Disease-free survival | |  |  |
|  | PD-L1 | 1.10 | 1.05 |
|  | MMR | 1.84 | 1.36 |
|  | TMB | 1.90 | 1.38 |
|  | Lauren’s classification | 1.24 | 1.05 |

VIF, Variance inflation factor; GVIF, Generalized VIF.

**Supplementary Table 4. Treatment-related adverse events.**

| **Terms** | **Grade 1-2** | **Grade 3-4** |
| --- | --- | --- |
| All [n (%)] | 73 (100.0) | 12 (16.4) |
| Myelosuppression [n (%)] | 46 (63.0) | 9 (12.3) |
| Anorexia [n (%)] | 46 (63.0) | 2 (2.7) |
| Anemia [n (%)] | 44 (60.3) | 6 (8.2) |
| Nausea [n (%)] | 43 (58.9) | 2 (2.7) |
| Fatigue [n (%)] | 40 (54.8) | 1 (1.4) |
| RCCEP [n (%)] | 22 (30.1) | 0 (0.0) |
| Neutropenia [n (%)] | 19 (26.0) | 4 (5.5) |
| Leukopenia [n (%)] | 17 (23.3) | 1 (1.4) |
| lymphopenia [n (%)] | 17 (23.3) | 0 (0.0) |
| Diarrhea [n (%)] | 14 (19.2) | 0 (0.0) |
| Infusion reaction [n (%)] | 12 (16.4) | 0 (0.0) |
| Rash [n (%)] | 12 (16.4) | 0 (0.0) |
| Hyperthyroidism [n (%)] | 12 (16.4) | 0 (0.0) |
| Thrombocytopenia [n (%)] | 9 (12.3) | 0 (0.0) |
| AST elevation [n (%)] | 8 (11.0) | 0 (0.0) |
| H[yponatremia [n (%)]](file:///C:\\Users\\xu%20xu%20xu\\AppData\\Local\\youdao\\dict\\Application\\9.2.0.0\\resultui\\html\\index.html%23\\javascript:;) | 8 (11.0) | 0 (0.0) |
| Hypothyroidism [n (%)] | 8 (11.0) | 0 (0.0) |
| Headache [n (%)] | 6 (8.2) | 0 (0.0) |
| ALT elevation [n (%)] | 6 (8.2) | 0 (0.0) |
| Hypokalemia [n (%)] | 3 (4.1) | 1 (1.4) |
| [Pneumonia [n (%)]](file:///C:\\Users\\xu%20xu%20xu\\AppData\\Local\\youdao\\dict\\Application\\9.2.0.0\\resultui\\html\\index.html%23\\javascript:;) | 1 (1.4) | 0 (0.0) |

∗Data presented as number (percentage) and graded according to the Common Terminology Criteria for Adverse Events (CTCAE), version 5.0. TRAEs, treatment-related adverse events.

**Supplementary Table 5. Postoperative complications.**

|  | **All population (n = 63)** | |
| --- | --- | --- |
|  | Grade 1-2 | Grade 3-4 |
| All [n (%)] | 63 (100.0) | 2 (3.2) |
| Pain [n (%)] | 40 (63.5) | 0 (0.0) |
| Fever [n (%)] | 21 (33.3) | 0 (0.0) |
| Pleural effusion [n (%)] | 14 (22.2) | 1 (1.6) |
| Pneumonia [n (%)] | 14 (22.2) | 0 (0.0) |
| Pulmonary atelectasis [n (%)] | 10 (15.9) | 0 (0.0) |
| Fistula [n (%)] | 1 (1.6) | 2 (3.2) |
| Haematemesis [n (%)] | 1 (1.6) | 0 (0.0) |
| Gastroplegia [n (%)] | 1 (1.6) | 0 (0.0) |
| Ileus [n (%)] | 1 (1.6) | 0 (0.0) |

∗Data presented as number (percentage) and graded according to the Clavien-Dindo classification.
